# Unraveling multi-state molecular dynamics in single-molecule FRET experiments. I. Theory of FRET-lines

**DOI:** 10.1063/5.0089134

**Published:** 2022-04-12

**Authors:** Anders Barth, Oleg Opanasyuk, Thomas-Otavio Peulen, Suren Felekyan, Stanislav Kalinin, Hugo Sanabria, Claus A. M. Seidel

**Affiliations:** 1 Institut für Physikalische Chemie, Lehrstuhl für Molekulare Physikalische Chemie, Heinrich-Heine-Universität, Düsseldorf, Germany; 2 Department of Physics and Astronomy, Clemson University, Clemson, South Carolina 29631, USA

## Abstract

Conformational dynamics of biomolecules are of fundamental importance for their function. Single-molecule studies of Förster Resonance Energy Transfer (smFRET) between a tethered donor and acceptor dye pair are a powerful tool to investigate the structure and dynamics of labeled molecules. However, capturing and quantifying conformational dynamics in intensity-based smFRET experiments remains challenging when the dynamics occur on the sub-millisecond timescale. The method of multiparameter fluorescence detection addresses this challenge by simultaneously registering fluorescence intensities and lifetimes of the donor and acceptor. Together, two FRET observables, the donor fluorescence lifetime τ_D_ and the intensity-based FRET efficiency *E*, inform on the width of the FRET efficiency distribution as a characteristic fingerprint for conformational dynamics. We present a general framework for analyzing dynamics that relates average fluorescence lifetimes and intensities in two-dimensional burst frequency histograms. We present parametric relations of these observables for interpreting the location of FRET populations in *E*–τ_*D*_ diagrams, called FRET-lines. To facilitate the analysis of complex exchange equilibria, FRET-lines serve as reference curves for a graphical interpretation of experimental data to (i) identify conformational states, (ii) resolve their dynamic connectivity, (iii) compare different kinetic models, and (iv) infer polymer properties of unfolded or intrinsically disordered proteins. For a simplified graphical analysis of complex kinetic networks, we derive a moment-based representation of the experimental data that decouples the motion of the fluorescence labels from the conformational dynamics of the biomolecule. Importantly, FRET-lines facilitate exploring complex dynamic models via easily computed experimental observables. We provide extensive computational tools to facilitate applying FRET-lines.

## I. INTRODUCTION

Many experimental techniques provide information on biomolecular structural heterogeneity and can be utilized to resolve ensembles of structures through integrative modeling.^1^ However, few techniques simultaneously inform on the structure and dynamics from picoseconds to seconds and offer the option for live-cell and *in vivo* measurements. Current advanced fluorescence spectroscopy has a broad dynamic range and can inform on local motions (femtosecond to nanosecond timescales), chain dynamics in disordered systems (nanoseconds to microseconds), and large-scale conformational changes (milliseconds to seconds),^2–4^ and it can be applied to a variety of *in vitro*, in live cells,^5–8^ and *in vivo* samples.^9^ Thus, there is considerable interest to exploit fluorescence spectroscopic information for integrative modeling of biological processes.^3,10^

A typical fluorescence spectroscopic modality is single-molecule Förster resonance energy transfer (smFRET). smFRET studies opened the possibility to interrogate the structure and conformational dynamics of individual fluorescently labeled biomolecules directly by the distance-dependent dipolar coupling of fluorophores,^11–15^ provided mechanistic insights in diverse areas of biological research, and could pave the way toward dynamic structural biology.^2^ Examples of biomolecular processes studied by smFRET are folding and unfolding transitions,^16–19^ dynamics of intrinsically disordered proteins,^20–24^ conformational dynamics of nucleic acids^25–28^ and proteins,^29–32^ multidomain structural rearrangements,^33,34^ and membrane receptors.^35,36^ The need for accurate and precise distance information for integrative modeling motivated a previous inter-laboratory benchmark study^37^ and the current effort of the smFRET community to establish standards for accurate processing of smFRET data.^38^ However, as will be exemplified in this manuscript, the complex conformational dynamics of multi-state systems with fast exchange kinetics can be overlooked. To this end, we generalize our previous approach that jointly interprets different spectroscopic observables to detect conformational dynamics^39^ to a general framework to highlight conformational dynamics and facilitate the interpretation of smFRET data of dynamic multi-state systems.

In smFRET experiments, a broad range of fluorescence spectroscopic observables, such as absorption and emission spectra,^40^ brightness and quantum yields,^41–43^ fluorescence lifetimes,^44,45^ and anisotropies,^12,46^ can be registered. However, the most used quantifier for FRET is the FRET efficiency, *E*, which is usually estimated by average fluorescence intensities. The FRET efficiency is the yield of the FRET process, i.e., the fraction of excited donor molecules that transfer energy to an acceptor molecule due to dipolar coupling. Besides intensities, fluorescence spectroscopy offers the anisotropy and the time-evolution information as quantifiers for FRET.^11,47–49^ Here, we provide a simple framework that combines information from fluorescence intensities and time-resolved observables. While we focus on revealing and interpreting conformational dynamics in smFRET experiments, our framework can be, in principle, applied to all FRET experiments where intensity and time-resolved information are registered simultaneously, such as fluorescence lifetime imaging (FLIM).

SmFRET experiments are performed on either freely diffusing molecules or molecules tethered to surfaces. In experiments on freely diffusing molecules, the molecules are excited and detected by confocal optics with point detectors.^50^ In experiments on surface-immobilized molecules, the molecules are typically excited by total internal reflection fluorescence (TIRF) and detected by cameras.^51^ The readout time limits the time resolution in camera-based detection to ∼1–10 ms.^52^ When paired with time-correlated single-photon counting (TCSPC), point detectors offer a significantly higher time-resolution with picosecond timing precision that enables accurate measurements of the fluorescence lifetimes. Fluorescence spectroscopy provides multidimensional observables that can be registered in parallel. A parallel spectral-, polarized-, and time-resolved registration of photons is called MFD (multiparameter fluorescence detection). Simultaneous registration of multiple fluorescence parameters by MFD has been widely applied to study the conformational dynamics of biomolecules in our and other groups.^50,53–59^

Due to its time resolution, confocal detection is particularly well-suited to study fast biomolecular dynamics. Various approaches have been developed to reveal and quantify dynamics by analyzing fluorescence intensities in confocal smFRET experiments. Different maximum likelihood approaches take advantage of the color information and the arrival time of single photons to determine kinetic rates from the unprocessed photon streams.^60,61^ An analysis of FRET efficiency histograms (FEHs) of single molecules reveals and informs on single-molecule kinetics. By variation of the integration time, dynamics are identified by changes of the FEH shapes.^39,62,63^ FEHs can be described by a combination of Gaussian distributions to reveals kinetic rate constants.^64^ For more accurate analysis, the shot noise in FEHs is explicitly accounted for in (dynamic) photon distribution analysis (PDA).^39,65^ Alternatively, variance analysis of the FRET efficiencies of single molecules reveals heterogeneities, e.g., by comparing the average photon arrival times in the donor and FRET channels.^56,66^ In burst variance analysis (BVA), the variance of the FRET efficiency is estimated, and dynamics are detected if the variance exceeds the shot noise limit.^67^ The two-channel kernel density estimator (FRET-2CDE filter) method applies a similar approach to detect anticorrelated fluctuations of the donor and acceptor signal.^68^ Finally, very fast conformational dynamics on the sub-millisecond timescale can be determined by fluorescence correlation spectroscopy,^15,69^ where the donor and FRET-sensitized acceptor fluctuations in the signal result in a characteristic anti-correlation in the cross-correlation function.^15,32^ For a robust estimation of the timescales of exchange, the contrast in fluorescence correlation spectroscopy (FCS) can be amplified in filtered-FCS by statistical filters that use spectral, lifetime, and anisotropy information registered in MFD experiments.^70,71^

**FIG. 1.**
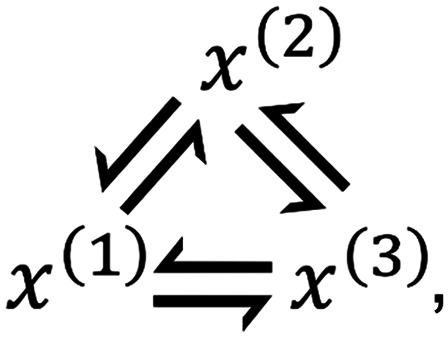
Overview of the described systems. The concept of FRET-lines is introduced in Secs. III A–III F. The static and dynamic FRET-lines for dyes whose position is fixed are discussed in Secs. III C, with the special cases of multistate systems and multi-exponential donor fluorescence decays being discussed in Secs. III G and IV A, respectively. The theory is then extended to covalently coupled dyes with long (∼20 Å) linkers in Secs. IV B and IV C. In Sec. IV D, FRET-lines for disordered systems are derived using standard polymer models, and order–disorder transitions (e.g., between folded and unfolded peptide chains) are discussed.

Even though various analysis methods have been developed for intensity-based FRET experiments, interpreting the data of systems with fast kinetics remains challenging. Two factors contribute to the problem. First, many analysis approaches require the kinetic model to be defined *a priori*. Second, most analysis methods are applied to reduced, one-dimensional representations of the experimental data, such as the FEH, the fluorescence decay, or the correlation function, which alone often do not provide sufficient information to distinguish between competing models. Hence, the model selection often remains ambiguous on the level of the individual data representations while also being the deciding factor for the correct interpretation and quantification of the observed dynamics. A solution to this problem is to exploit the multidimensionality of the smFRET data in MFD experiments, where it has early been recognized that conformational dynamics could be detected in two-dimensional burst frequency histograms of the FRET efficiency, *E*, and donor fluorescence lifetime, τ_*D*_.^72^ The different averaging behavior of the two observables produces distinct dynamic fingerprints in the two-dimensional plots. To describe these patterns, parametric relationships have been introduced to describe static molecules,^72^ those undergoing dynamic exchange between two^39^ or three^73^ states, folding–unfolding transitions,^74,75^ and disordered systems described by idealized polymer models.^20^ Here, we call the guidelines defined by these parametric relationships “FRET-lines.” Despite their wide application by expert users, there is a lack of a comprehensive overview and description of a general formalism to compute FRET-lines, especially if experimental complications, such as the dynamics of the flexibly coupled fluorophores, have to be considered. We present a detailed discussion of the different theoretical and practical aspects of FRET-lines. To interpret two-dimensional burst frequency histograms computed using average intensities and lifetimes, we first introduce the average observables and relate them to conformational heterogeneities (Fig. 1, Concepts). Using a simple two-state system, we describe how model parameters can be recovered from the FRET-lines. Next, we present the definition of the FRET-lines and provide a rigorous framework for their calculation. We present transformations that can be applied to experimental data that directly visualize conformational heterogeneity and can be used to resolve the species population of exchanging states and generalize the concepts presented for two-state systems to multi-state systems (Fig. 1, Concepts). The second part of this manuscript assembles the most relevant equations needed to interpret data of static and dynamic systems for dyes that are fixed stiffly to the molecule of interest (Fig. 1, Fixed dyes), and presents conformational heterogeneity caused by flexibly coupling dyes is accounted for (Fig. 1, Flexible dyes). Finally, we present FRET-lines that can inform on an order-disorder transition (Fig. 1, Disordered systems).

## II. THEORY

### A. Förster resonance energy transfer

FRET is the non-radiative energy transfer from an excited donor (*D*) to an acceptor (*A*) fluorophore by dipolar coupling that depends strongly on the interdye distance *R*_*DA*_. The rate constant of the energy transfer from *D* to *A*, *k*_*RET*_, depends on the distance between the donor and the acceptor transition dipole moments,^11^

$$k_{RET}=\frac{k_{F,D}}{\Phi_{F,D}}{\left(\frac{R_{0}}{R_{DA}}\right)}^{6}=\frac{1}{\tau_{D\left(0\right)}}{\left(\frac{R_{0}}{R_{DA}}\right)}^{6},$$ (1)

where *k*_*F*,*D*_ is the radiative rate constant of the donor, Φ_*F*,*D*_ is the fluorescence quantum yield of the donor, *R*_0_ is the dye-pair specific Förster radius, and *R*_*DA*_ is the *DA*-distance. The Förster radius, *R*_0_, depends on the mutual orientation of the fluorophore dipoles, captured by the orientation factor *κ*^2^. Moreover, *R*_0_ depends on the spectral overlap integral $J\left(\lambda \right)$, the refractive index of the medium, *n*, and Φ_*F*,*D*_, the quantum yield of the donor fluorophore,

$$R_{0}^{6}=\frac{9\left(\ln\;10\right)}{128\pi^{5}\cdot N_{A}}\cdot \frac{\kappa^{2}\Phi_{F,D}J\left(\lambda \right)}{n^{4}}.$$ (2)

Here, *N*_*A*_ is Avogadro’s constant. The spectral overlap integral is defined by $J\left(\lambda \right)=\int f_{D}\left(\lambda \right)\varepsilon_{A}\left(\lambda \right)\lambda^{4}d\lambda$, where $f_{D}\left(\lambda \right)$ is the normalized emission spectrum of the donor and $\varepsilon_{A}\left(\lambda \right)$ is the extinction of the acceptor at wavelength *λ*. For simplicity, we focus on dyes that reorient fast compared to the FRET rate constant. For such a case, *κ*^2^ can be approximated by the isotropic average, ${\left\langle \kappa^{2}\right\rangle }_{iso}=2/3$. This approximation applies to freely rotating dyes that are flexibly coupled to biomolecules via long linkers.^33,37,76,77^

The rate constant of FRET and the resulting fluorescence lifetimes relate to its FRET efficiency, *E*, by

$$E=\frac{k_{RET}}{k_{RET}+k_{F,D}+\sum_{j}k_{Q}^{\left(j\right)}}=1-\frac{\tau_{D\left(A\right)}}{\tau_{D\left(0\right)}}.$$ (3)

Here, $\sum_{j}k_{Q}^{\left(j\right)}$ is the sum over the rate constants of all additional non-radiative pathways depopulating the excited state of the donor, and *τ*_*D*(0)_ and *τ*_*D*(*A*)_ are the donor fluorescence lifetimes in the absence and presence of the acceptor. The FRET efficiency is related to the interdye distance, *R*_*DA*_, by^11^

$$E=\frac{1}{1+{\left(\frac{R_{DA}}{R_{0}}\right)}^{6}}.$$ (4)

Thus, the fluorescence lifetime of the donor in the presence of the acceptor is related to the FRET efficiency and the interdye distance by

$$\tau_{D\left(A\right)}=\left(1-E\right)\tau_{D\left(0\right)}=\frac{\tau_{D\left(0\right)}}{1+{\left(\frac{R_{0}}{R_{DA}}\right)}^{6}}.$$ (5)

### B. Time-resolved fluorescence

In single-molecule FRET experiments with pulsed excitation, the detected photons are also characterized by their delay time with respect to the excitation pulse. The distribution of delay times *t* of photons emitted by a donor with a fluorescence lifetime *τ*_*D*(0)_ that is quenched by FRET with a rate constant, *k*_*FRET*_, follows an exponential decay,

$$f_{D|D}^{\left(DA\right)}\left(t\right)=k_{F,D}e^{-t/\tau_{D\left(A\right)}}\;with\;\tau_{D\left(A\right)}=1/\left(\tau_{D\left(0\right)}^{-1}+k_{FRET}\right),$$ (6)

where *τ*_*D*(*A*)_ is the fluorescence lifetime of the donor in the presence of FRET. Here, the superscript of the time-resolved fluorescence signal $f_{D|D}^{\left(DA\right)}$ denotes that the sample is a FRET sample, and the subscript indicates that the fluorescence is measured in the donor channel after donor excitation. If the radiative rate constant, *k*_*F*,*D*_, is independent of the FRET rate, the fluorescence decay of a mixture of species with different fluorescence lifetimes with the lifetime distribution *p*(*τ*_*D*(*A*)_) is given by

$$f_{D|D}^{\left(DA\right)}\left(t\right)=k_{F,D}\int p\left(\tau_{D\left(A\right)}\right)e^{-\frac{t}{\tau_{D\left(A\right)}}}d\tau_{D\left(A\right)},$$ (7)

where $f_{D|D}^{\left(DA\right)}\left(t\right)$ corresponds to the Laplace transform of the distribution of fluorescence lifetimes. Equation (7) can also be expressed in terms of the interdye distance, *R*_*DA*_, directly as

$$f_{D|D}^{\left(DA\right)}\left(t\right)=k_{F,D}\int p\left(R_{DA}\right)e^{-\frac{t}{\tau_{D\left(0\right)}}\left[1+{\left(\frac{R_{0}}{R_{DA}}\right)}^{6}\right]}dR_{DA}.$$ (8)

These equations highlight the potential to resolve the conformational heterogeneity in terms of the distribution of the interdye distance *p*(*R*_*DA*_) from the FRET-sensitized donor fluorescence decay. The interpretation hereby depends on the choice of the model function for *p*(*R*_*DA*_); thus, it is important to consider the broadening introduced by the flexible linkers, which will be discussed in detail in Sec. IV B.

In this work, it is assumed that the properties of the fluorophores do not vary for different conformational states of the molecule (homogeneous approximation). In practice, this assumption does often not hold when the environment of the fluorophores changes, leading to local quenching by aromatic residues, spectral shifts, or sticking interactions with the biomolecular surface. For details on how to account for a correlation between the photophysical and conformational states, the reader is referred to Ref. 48.

### C. Intensity-based observable: FRET efficiency

The FRET efficiency can be quantified either from the number of photons emitted by the acceptor dyes due to FRET or from the decrease in the number of photons emitted by the donor dye due to the transfer of energy to the acceptor. Using the fluorescence intensities *F* that is fully corrected for the quantum yields and detection efficiencies, the FRET efficiency is given by

$$E=\frac{F_{A|D}^{\left(DA\right)}}{F_{A|D}^{\left(DA\right)}+F_{D|D}^{\left(DA\right)}}=\frac{F_{D|D}^{\left(D0\right)}-F_{D|D}^{\left(DA\right)}}{F_{D|D}^{\left(D0\right)}}.$$ (9)

The superscripts refer to the sample type (*DA* is a FRET sample), and the subscripts refer to the excitation, (…|*X*), and detection, (*X*|…), channels. *D* and *A* refer to the donor and acceptor fluorophore, respectively. For instance, $F_{A|D}^{\left(DA\right)}$ is the fluorescence intensity of the acceptor ($\left.A|\ldots \right)$ of a FRET molecule (*DA*), given that the donor was excited (…|*D*). In practice, the detected raw signals in the donor, (*I*_*D*|*D*_), and acceptor, (*I*_*A*|*D*_), channels need to be corrected (for details, see Ref. 37) to yield fluorescence intensities, *F*.

In a time-resolved experiment, the fluorescence intensity *F* is determined by integrating the fluorescence intensity decay $f\left(t\right)$. For the distribution of fluorescence lifetimes, *p*(*τ*_*D*(*A*)_), the integrated donor fluorescence intensity is given by

$$F_{D|D}^{\left(DA\right)}=k_{F,D}\int \int p\left(\tau_{D\left(A\right)}\right)e^{-\frac{t}{\tau_{D\left(A\right)}}}d\tau_{D\left(A\right)}dt.$$ (10)

The integral over *t* is equivalent to the fluorescence lifetime, $\int e^{-t/\tau_{D\left(A\right)}}dt=\tau_{D\left(A\right)}$, and the fluorescence intensity is, hence, proportional to the species-averaged fluorescence lifetime, $\left\langle {\tau_{D\left(A\right)}}_{x}\right\rangle$,

$$F_{D|D}^{\left(DA\right)}=k_{F,D}\int \tau_{D\left(A\right)}p\left(\tau_{D\left(A\right)}\right)d\tau =k_{F,D}{\left\langle \tau_{D\left(A\right)}\right\rangle }_{x}.$$ (11)

Through the definition of the FRET efficiency from the photon counts of the donor fluorophore in the presence and absence of FRET, $F_{D|D}^{\left(DA\right)}$ and $F_{D|D}^{\left(D0\right)}$ [Eq. (9)], we can relate the intensity-averaged FRET efficiency *E* to the time-resolved fluorescence decays of the donor in the presence and absence of FRET, $f_{D|D}^{\left(DA\right)}\left(t\right)$ and $f_{D|D}^{\left(D0\right)}\left(t\right)$,

$$E=1-\frac{k_{F\cdot D}\int f_{D|D}^{\left(DA\right)}\left(t\right)dt}{k_{F,D}\int f_{D|D}^{\left(D0\right)}\left(t\right)dt}=1-\frac{{\left\langle \tau_{D\left(A\right)}\right\rangle }_{x}}{{\left\langle \tau_{D\left(0\right)}\right\rangle }_{x}}.$$ (12)

For now, we consider the case of a single-exponential donor lifetime, that is, ${\left\langle \tau_{D\left(0\right)}\right\rangle }_{x}=\tau_{D\left(0\right)}$, and consider the effect of multi-exponential donor fluorescence lifetimes in Sec. IV A.

### D. Lifetime-based observable: Average delay time

In smFRET experiments with pulsed excitation, the detected photons are characterized by their delay time with respect to the excitation pulse. Due to the limited number of photons available in a single-molecule experiment, it is impossible to recover the distribution of fluorescence lifetimes *p*(*τ*_*D*(*A*)_). However, an average delay time, $\left\langle t\right\rangle$, can be determined reliably.

The average delay time $\left\langle t\right\rangle$ is defined by

$$\left\langle t\right\rangle =\int t\cdot p_{D|D}^{\left(DA\right)}\left(t\right)dt=\frac{\int t\cdot f_{D|D}^{\left(DA\right)}\left(t\right)dt}{\int f_{D|D}^{\left(DA\right)}\left(t\right)dt},$$ (13)

where $p_{D|D}^{\left(DA\right)}\left(t\right)$ is the normalized fluorescence decay that describes the probability distribution of delay times. For a distribution of fluorescence lifetimes *p*(*τ*_*D*(*A*)_), the average $\left\langle t\right\rangle$ is then

$$\begin{array}{ll} \left\langle t\right\rangle & =\frac{\int t\cdot \left[\int p\left(\tau_{D\left(A\right)}\right)e^{-t/\tau_{D\left(A\right)}}d\tau_{D\left(A\right)}\right]dt}{\int \int p\left(\tau_{D\left(A\right)}\right)e^{-t/\tau_{D\left(A\right)}}d\tau_{D\left(A\right)}dt}\\ & =\frac{\int p\left(\tau_{D\left(A\right)}\right)\left[\int t\cdot e^{-t/\tau_{D\left(A\right)}}dt\right]d\tau_{D\left(A\right)}}{\int p\left(\tau_{D\left(A\right)}\right)\left[\int e^{-t/\tau_{D\left(A\right)}}dt\right]d\tau_{D\left(A\right)}}. \end{array}$$ (14)

The inner integrals are given by $\int e^{-t/\tau_{D\left(A\right)}}dt=\tau_{D\left(A\right)}$ and $\int t\cdot e^{-t/\tau_{D\left(A\right)}}dt=\tau_{D\left(A\right)}^{2}$, resulting in the following expression for the average delay time:

$$\left\langle t\right\rangle \to \frac{\int \tau_{D\left(A\right)}^{2}p\left(\tau_{D\left(A\right)}\right)d\tau_{D\left(A\right)}}{\int \tau_{D\left(A\right)}p\left(\tau_{D\left(A\right)}\right)d\tau_{D\left(A\right)}}=\frac{\bar{\tau_{D\left(A\right)}^{2}}}{\bar{\tau_{D\left(A\right)}}},$$ (15)

where $\bar{\tau_{D\left(A\right)}}$ and $\bar{\tau_{D\left(A\right)}^{2}}$ are the first and second moments of the lifetime distribution, respectively. Thus, in an ideal measurement (i.e., in the absence of shot noise or other experimental imperfections), the average delay time $\left\langle t\right\rangle$ converges to the ratio of the second and first moments of the lifetime distribution. Importantly, the average delay time $\left\langle t\right\rangle$ informs on the second moment $\bar{\tau_{D\left(A\right)}^{2}}$ of the fluorescence lifetime distribution, which conveys information about its variance.

It is important to note that the average delay time $\left\langle t\right\rangle$ is equivalent to the intensity-weighted average fluorescence lifetime, which we denote by ${\left\langle \tau_{D\left(A\right)}\right\rangle }_{F}$ to distinguish it from the species-weighted average fluorescence lifetime, ${\left\langle \tau_{D\left(A\right)}\right\rangle }_{x}$, introduced above. Note that the fluorescence intensity, *F*(*τ*_*D*(*A*)_), of a species with a lifetime *τ*_*D*(*A*)_ is proportional to its fluorescence lifetime,

$$F\left(\tau_{D\left(A\right)}\right)=p\left(\tau_{D\left(A\right)}\right)\int k_{F,D}e^{-\frac{t}{\tau_{D\left(A\right)}}}dt=k_{F,D}\tau_{D\left(A\right)}p\left(\tau_{D\left(A\right)}\right).$$ (16)

Then, the intensity-weighted average lifetime, ${\left\langle \tau_{D\left(A\right)}\right\rangle }_{F}$, is given by

$$\begin{array}{ll} {\left\langle \tau_{D\left(A\right)}\right\rangle }_{F} & =\frac{\int F\left(\tau_{D\left(A\right)}\right)\tau_{D\left(A\right)}d\tau_{D\left(A\right)}}{\int F\left(\tau_{D\left(A\right)}\right)d\tau_{D\left(A\right)}}\\ & =\frac{\int_{0}^{\infty }\tau_{D\left(A\right)}^{2}p\left(\tau_{D\left(A\right)}\right)d\tau_{D\left(A\right)}}{\int_{0}^{\infty }\tau_{D\left(A\right)}p\left(\tau_{D\left(A\right)}\right)d\tau_{D\left(A\right)}}=\frac{\bar{\tau_{D\left(A\right)}^{2}}}{\bar{\tau_{D\left(A\right)}}}, \end{array}$$ (17)

which is equivalent to the result for the above-mentioned average delay time.

So far, we have assumed that the fluorescence is excited by an ideal *δ*-pulse. Experimentally, the analysis is complicated due to the finite width of the laser excitation pulse and characteristics of the detection electronics, defining the instrument response function (IRF). In the analysis, the IRF is accounted for by convolution with the ideal decay model. For low photon numbers, accurate lifetimes are best extracted using a maximum likelihood estimator (MLE) that correctly accounts for the noise characteristics of the photon detection, anisotropy effects, and the presence of the background signal.^78^ The fluorescence lifetime obtained by maximizing the likelihood function is equivalent to the intensity-averaged fluorescence lifetime, i.e., $\tau_{MLE}={\left\langle \tau_{D\left(A\right)}\right\rangle }_{F}$ (see the supplementary material, Note 1).

## III. CONCEPTS

### A. Detecting dynamics using the fluorescence lifetime information

**FIG. 2.**
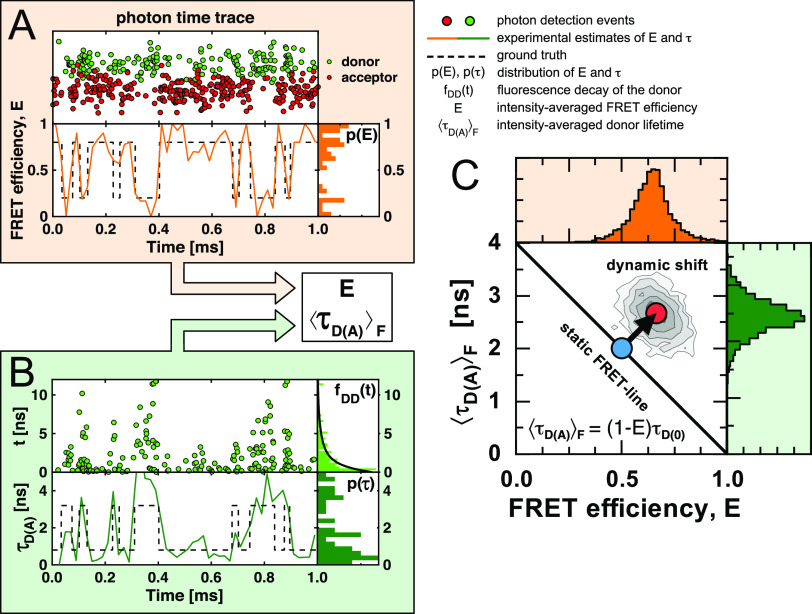
Identifying conformational dynamics and heterogeneities in single-molecule FRET. (a) Simulated single-molecule interconverting between states with different FRET efficiencies (dashed line). The molecule emits red and green photons (circles) registered in the donor and acceptor (FRET) channel (top). An analysis of the photons yields an estimate of the FRET efficiency (orange line) with the corresponding distribution of average FRET efficiencies visualized as a histogram to the right. (b) Time-correlated single-photon counting additionally measures the time ***t*** since the excitation pulse for each photon (top left), from which the fluorescence lifetime of the donor, $\tau_{D\left(A\right)}$, is estimated (bottom left). In practice, only the intensity weighted average fluorescence lifetime ${\left\langle \tau_{D\left(A\right)}\right\rangle }_{F}$ can be estimated from the histogram of delay times (top right). The time trace of the fluorescence lifetimes (green line, bottom) and the distribution of fluorescence lifetimes (bottom right) are not accessible. (c) Single-molecule histogram of ***E*** and ${\left\langle \tau_{D\left(A\right)}\right\rangle }_{F}$. A shift from the static FRET-line defined by ${\left\langle \tau_{D\left(A\right)}\right\rangle }_{F}=\left(1-E\right)\tau_{D\left(0\right)}$ highlights heterogeneities in the FRET efficiency and indicates conformational dynamics.

The detection and analysis of fast conformational dynamics in smFRET experiments remains challenging due to the limited signal collected for each single molecule event [Fig. 2(a)]. Here, kinetics is considered fast if the associated exchange of states happens on a timescale comparable to or faster than the observation time. In confocal experiments, the upper limit of the observation time is set by the diffusion time of a molecule in the confocal volume. The photon detection rate determines the lower limit of the observation time. In a typical confocal smFRET experiment, usually less than 500 photons are detected per single molecule in an observation time of a few milliseconds. For each molecule, the FRET efficiency, *E*, is calculated from the integrated fluorescence intensities. As most a few hundred photons are registered, only an average FRET efficiency, *E*, can be estimated reliably for each molecule, and the kinetic information is partially lost [Fig. 2(a)].

In experiments with time-correlated single-photon counting in addition to the fluorescence intensity and the delay time $\left\langle t\right\rangle$, the excitation pulse is recorded for each registered photon. The average delay time $\left\langle t\right\rangle$ relates to the fluorescence lifetime *τ*_*D*(*A*)_. Importantly, *t* corresponds to the intensity weighted average fluorescence lifetime ${\left\langle \tau_{D\left(A\right)}\right\rangle }_{F}$. The fluorescence lifetime of the donor in the presence of FRET fluctuates with the FRET efficiency [Fig. 2(b)]. For a donor dye with a mono-exponential fluorescence decay and a fixed distance between the dyes, the quantities *E*, *τ*_*D*(*A*)_ and ${\left\langle \tau_{D\left(A\right)}\right\rangle }_{F}$ are related as follows:

$$\begin{array}{c} E=1-\frac{\tau_{D\left(A\right)}}{\tau_{D\left(0\right)}},\;\left\langle t\right\rangle ={\left\langle \tau_{D\left(A\right)}\right\rangle }_{F}=\tau_{D\left(A\right)}, \end{array}$$ (18)

where $\tau_{D\left(0\right)}$ is the fluorescence lifetime of the donor in the absence of FRET. In this case, the two observables *E* and ${\left\langle \tau_{D\left(A\right)}\right\rangle }_{F}$ follow the linear dependence: ${\left\langle \tau_{D\left(A\right)}\right\rangle }_{F}=\tau_{D\left(0\right)}\left(1-E\right)$. We call the reference line described by this relation the ideal static FRET-line, as it is valid for single molecules and ensembles with a single FRET efficiency.

When the molecule switches between different conformational states with different FRET efficiencies during the observation time, only average quantities can be estimated robustly due to the limited number of photons.^78^ In this case, the FRET efficiency relates to the species average of lifetimes ${\left\langle \tau_{D\left(A\right)}\right\rangle }_{x}$. On the other hand, the intensity-weighted average fluorescence lifetime ${\left\langle \tau_{D\left(A\right)}\right\rangle }_{F}$ is determined by the donor intensity, and species with a smaller FRET efficiency contribute more photons to the donor fluorescence decay. Therefore, the estimated average lifetime, $\left\langle t\right\rangle ={\left\langle \tau_{D\left(A\right)}\right\rangle }_{F}$, is biased toward longer fluorescence lifetimes compared to the species average ${\left\langle \tau_{D\left(A\right)}\right\rangle }_{x}$ [Fig. 1(b)],

$$\begin{array}{c} E=1-\frac{{\left\langle \tau_{D\left(A\right)}\right\rangle }_{x}}{\tau_{D\left(0\right)}},\;\left\langle t\right\rangle ={\left\langle \tau_{D\left(A\right)}\right\rangle }_{F}>{\left\langle \tau_{D\left(A\right)}\right\rangle }_{x}. \end{array}$$ (19)

Because *E* and ${\left\langle \tau_{D\left(A\right)}\right\rangle }_{F}$ correspond to different averages, the pair of experimental observables $\left(E,{\left\langle \tau_{D\left(A\right)}\right\rangle }_{F}\right)$ reveals sample dynamics and heterogeneities through a deviation from the ideal behavior. In single-molecule counting histograms of $\left(E,{\left\langle \tau_{D\left(A\right)}\right\rangle }_{F}\right)$, heterogeneities are identified by a shift of populations from the reference static FRET-line [Fig. 2(c)].

There are many ways to compute other reference lines that relate a FRET efficiency, *E*, to an average fluorescence weighted lifetime, ${\left\langle \tau_{D\left(A\right)}\right\rangle }_{F}$. We call any parametric relation between the FRET observables a “FRET-line.” FRET-lines can serve as valuable guides to interpret experimental distributions because they relate model parameters to experimental observables, identify dynamic populations, and allow to understand the dynamic exchange in complex kinetic networks encoded as an experimental fluorescence fingerprint.

### B. Detecting dynamics by intensity-based approaches

In purely intensity-based approaches, the average inter-photon time limits the ability to detect conformational dynamics. We demonstrate this limitation by simulations of smFRET experiments of molecules that undergo conformational dynamics between distinct states at increasing interconversion rates. We simulate typical smFRET experiments with a count rate per molecule of 100 kHz. The simulated smFRET data were processed using the popular burst variance analysis (BVA)^67^ procedure.

**FIG. 3.**
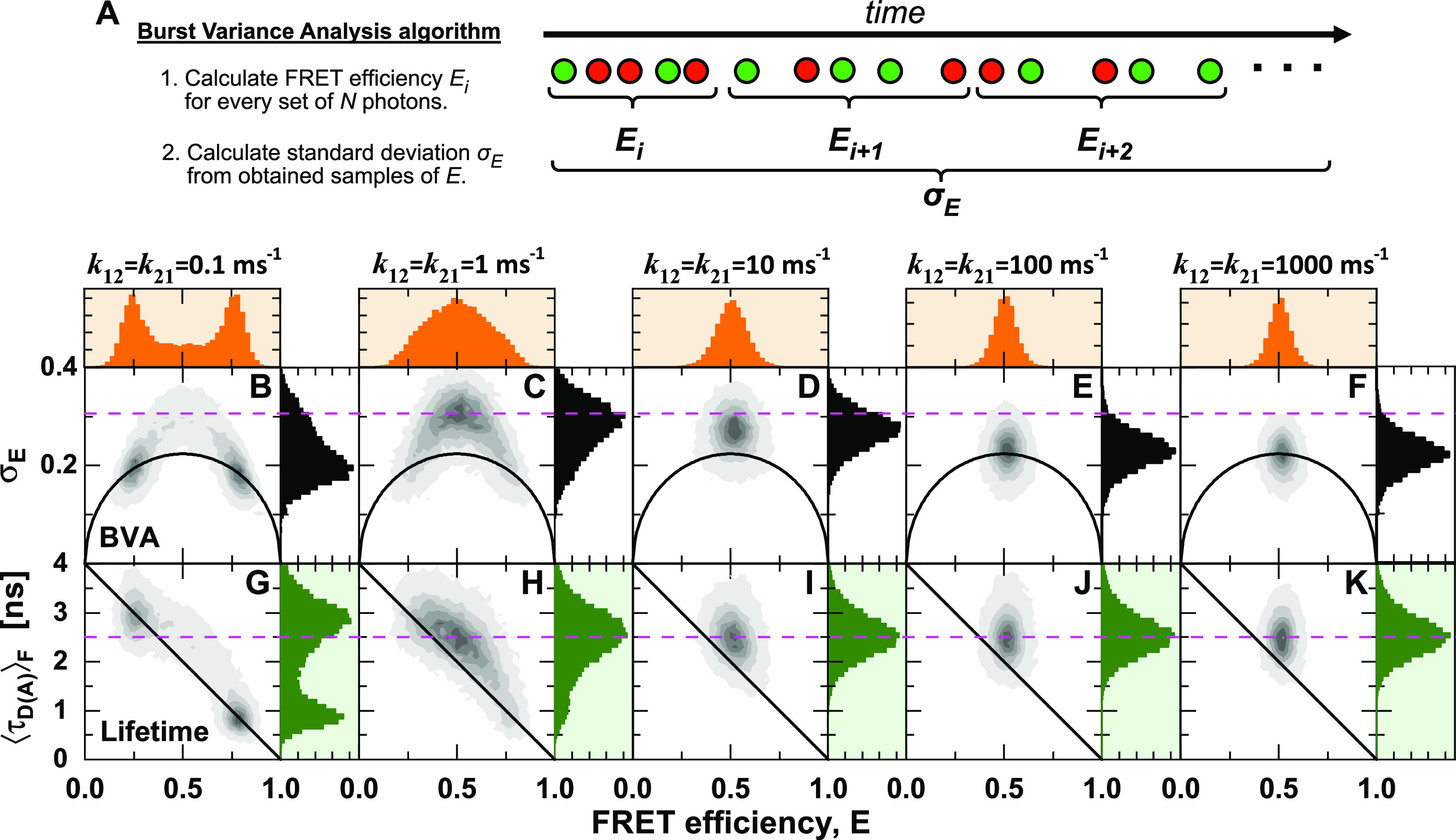
Comparison of intensity-based and lifetime-based indicators of dynamics. (a) Illustration of the algorithm used in burst variance analysis (BVA) to estimate the standard deviation of the FRET efficiency for a set of fluorescence photons of a single-molecule event. The photon trace is sub-sampled by the number of photons, *N* (typically *N* = 5). For every sub-sample, the FRET efficiency, *E*, is estimated. An estimate of the standard deviation of the FRET efficiency, *σ*_*E*_, is obtained by the estimates of *E*. (b)–(k) Molecule-wise histograms of simulated datasets with indicated interconversion rates between two states with FRET efficiencies of 0.25 and 0.80, corresponding to interdye distances of 60 and 40 Å at a Förster radius of 50 Å, and a diffusion time of 0.5 ms. (b)–(f) In BVA, conformational dynamics increase the standard deviation of the FRET efficiency *σ*_*E*_ beyond the expected shot noise variance (black line) given by Eq. (21). (g)–(k) The comparison of the two estimators of the FRET efficiency, *E* and ${\left\langle \tau_{D\left(A\right)}\right\rangle }_{F}$, reveals conformational dynamics as a shift from the static FRET-line (diagonal line) given by $E=1-\frac{{\left\langle \tau_{D\left(A\right)}\right\rangle }_{F}}{\tau_{D\left(0\right)}}$. BVA is most sensitive to slow dynamics, while the standard deviation of the FRET efficiency is underestimated at fast dynamics. In contrast, the lifetime-based indicator detects conformational dynamics irrespective of their timescale. The dashed magenta line indicates the expected position of the dynamic population on the y-axis. For BVA, a photon window of *N* = 5 was used.

In BVA, the variance of the FRET efficiency is estimated for every detected single molecule to reveal conformational dynamics happening on a timescale of the single-molecule burst duration. The basic idea of BVA is to obtain an estimate of the distribution of FRET efficiencies within a single-molecule event by sampling the FRET efficiency with a higher rate than the structural dynamics. In BVA, the FRET efficiency trace of single-molecule bursts is sampled [Fig. 3(a)], and the standard deviation of the FRET efficiency within a single-molecule burst is estimated by

$$\sigma_{E}=\sqrt{\frac{1}{M}\overset{M}{\underset{i=1}{\sum }}{\left(E_{i}-E\right)}^{2}}.$$ (20)

Here, *E*_*i*_ is the FRET efficiency of a sample, *M* is the total number of samples, and *E* is the average FRET efficiency of the single-molecule event obtained by Eq. (9). The standard deviation *σ*_*E*_ is then plotted against the average FRET efficiency *E* [Fig. 3(b)]. The lower boundary for the standard deviation of the FRET efficiency is given by the theoretical shot noise limit, determined by the number of photons per sample *N*,^79^

$$\sigma_{E}=\sqrt{\frac{E\left(1-E\right)}{N}}.$$ (21)

This above equation is the corresponding static FRET-line in BVA. Single-molecule events that exceed this limit are considered dynamic.

The simulated data were processed by BVA with a photon window of *N* = 5 [Figs. 3(b)–3(f)]. A standard deviation *σ*_*E*_ observed in BVA that exceeds the shot noise limit decreases as the dynamics become faster [Figs. 3(b)–3(f)]. In the simulations, the average inter-photon time was 10 *µ*s, and the time resolution is further reduced due to the need to average over a given photon number (typically, *N* = 5).^67^ All faster processes than this limit will be averaged over the sampling time and, thus, not detected as dynamic [Figs. 3(e) and 3(f)]. The dependency on the timescale of dynamics makes it difficult to predict the exact shape of the observed distributions, which requires taking into account the experimental photon count distribution.^67^ Hence, they have mainly been used as qualitative indicators of conformational dynamics. It should be noted that dynamics on timescales faster than the inter-photon time can still be detected by fluorescence correlation spectroscopy (FCS), wherein the effective time resolution is determined mainly by the signal-to-noise ratio. However, in contrast to the single-molecule analysis, it is challenging to directly identify states or their connectivity from the FCS curves.

On the other hand, using the relation between the FRET efficiency *E* and the intensity-averaged donor fluorescence lifetime ${\left\langle \tau_{D\left(A\right)}\right\rangle }_{F}$, conformational dynamics are identified even if they are fast [Figs. 3(g)–3(k)]. This lifetime-based indicator is independent of the detection count rate because it relies only on the deviation of the fluorescence decay from the ideal single-exponential behavior. Hence, all dynamic processes that are slower than the fluorescence lifetime (>ns) are detected, and no decrease of the dynamic shift is observed at increasing timescales of the dynamics. Combining fluorescence lifetimes and intensities is, thus, superior in detecting and visualizing fast conformational dynamics than approaches that rely on intensities alone.

In practice, one must consider potential artifacts that lead to a false-positive detection of dynamics. Examples include dark states of the acceptor (e.g., due triplet states). Acceptor dark states always affect intensity-based indicators of dynamics as they result in fluctuations of the apparent FRET efficiency. The effect of dark acceptor states on the donor fluorescence lifetime depends on the nature of the photophysical change. Triplet states often still act as FRET acceptors with a similar Förster radius as the single state; a similar situation is found for the *cis–trans* isomerization of cyanine dyes, such as Cy5.^80–82^ Radical or ionic dark states, on the other hand, often are not viable FRET acceptors. In this case, the donor lifetime will fluctuate as a function of the photophysical state of the acceptor.^83^

### C. FRET-lines of static and dynamic molecules

In addition to detecting the presence of dynamics, FRET-lines are a powerful tool to obtain information about the nature of the dynamic exchange and identify the limiting states and their connectivity. So far, we have introduced the static FRET-line that describes the ideal relationship between the fluorescence lifetime of the donor fluorophore and the FRET efficiency in the absence of dynamics. For the experimental observables *E* and ${\left\langle \tau_{D\left(A\right)}\right\rangle }_{F}$, the static FRET-line is defined as

$$static\;FRET-line:\;E=1-\frac{{\left\langle \tau_{D\left(A\right)}\right\rangle }_{F}}{\tau_{D\left(0\right)}}.$$ (22)

Importantly, this relationship only holds for a fixed distance between the dyes resulting in a single FRET rate, in which case the intensity-weighted average fluorescence lifetime is equal to the species average, ${\left\langle \tau_{D\left(A\right)}\right\rangle }_{F}={\left\langle \tau_{D\left(A\right)}\right\rangle }_{x}$. In the case of a distribution of distances (and hence lifetimes) that are sampled during the observation time, the intensity-averaged fluorescence lifetime is biased toward species with long fluorescence lifetimes and, thus, low FRET efficiencies, and ${\left\langle \tau_{D\left(A\right)}\right\rangle }_{F}>{\left\langle \tau_{D\left(A\right)}\right\rangle }_{x}$. This results in the shift of the populations from the static FRET-line [Fig. 3(c)]. We call this shift the “dynamic shift” and define it as the minimal distance to the static FRET-line for a given point in the *E*–${\left\langle \tau_{D\left(A\right)}\right\rangle }_{F}$ histogram.

We now consider the simplest case of dynamics wherein the molecule switches between two defined conformations during the observation time,

$$E^{\left(1\right)},\;\tau_{D\left(A\right)}^{\left(1\right)}\begin{array}{c} \overset{k_{12}}{⟶}\\ \underset{k_{21}}{⟵} \end{array}E^{\left(2\right)},\;\tau_{D\left(A\right)}^{\left(2\right)},$$ (23)

where *k*_12_ and *k*_21_ are the microscopic interconversion rates between the two states that define the probability that a molecule spends a fraction of time *x*^(*i*)^ in state *i* during the observation time. The fractions *x*^(*i*)^ are stochastic quantities and change from one observation to another. For now, we are not interested in the exact distribution of the state occupancy *x*^(1)^ and treat it as the independent parameter of the model. This is equivalent to the assumption of a uniform distribution for *x*^(1)^. The effect of the distribution of state occupancies is discussed in detail in Paper II.

Assuming that each state is characterized by the same donor fluorescence lifetime, the species-weighted and fluorescence-weighted average lifetimes depend only on the state occupancy of the individual states, *x*^(1)^ and *x*^(2)^ = 1 − *x*^(1)^,

$${\left\langle \tau_{D\left(A\right)}\right\rangle }_{x}=x^{\left(1\right)}\tau_{D\left(A\right)}^{\left(1\right)}+\left(1-x^{\left(1\right)}\right)\tau_{D\left(A\right)}^{\left(2\right)},$$ (24)

$${\left\langle \tau_{D\left(A\right)}\right\rangle }_{F}=\frac{{\left\langle \tau_{D\left(A\right)}^{2}\right\rangle }_{x}}{{\left\langle \tau_{D\left(A\right)}\right\rangle }_{x}}=\frac{x^{\left(1\right)}{\left(\tau_{D\left(A\right)}^{\left(1\right)}\right)}^{2}+\left(1-x^{\left(1\right)}\right){\left(\tau_{D\left(A\right)}^{\left(2\right)}\right)}^{2}}{x^{\left(1\right)}\tau_{D\left(A\right)}^{\left(1\right)}+\left(1-x^{\left(1\right)}\right)\tau_{D\left(A\right)}^{\left(2\right)}}.$$ (25)

Here, we changed the notation from the continuous distribution of lifetimes to the discrete case, that is,

$$p\left(\tau_{D\left(A\right)}\right)=\left\{\begin{array}{cc} x^{\left(1\right)}\; & for\;\tau_{D\left(A\right)}=\tau_{D\left(A\right)}^{\left(1\right)},\\ 1-x^{\left(1\right)}\; & for\;\tau_{D\left(A\right)}=\tau_{D\left(A\right)}^{\left(2\right)},\\ 0\; & otherwise. \end{array}\right.$$ (26)

To obtain a general relationship between the observables *E* and ${\left\langle \tau_{D\left(A\right)}\right\rangle }_{F}$, we find the line that describes all values of *x*^(1)^ by combining Eqs. (24) and (25), relating the species-weighted average lifetime to the intensity-weighted average lifetime,

$$\begin{array}{ll} dynamic\;FRET-line:E & =1-\frac{{\tau_{D\left(A\right)}}_{x}}{\tau_{D\left(0\right)}}\\ & =1-\frac{1}{\tau_{D\left(0\right)}}\cdot \left[\frac{\tau_{D\left(A\right)}^{\left(1\right)}\cdot \tau_{D\left(A\right)}^{\left(2\right)}}{\tau_{D\left(A\right)}^{\left(1\right)}+\tau_{D\left(A\right)}^{\left(2\right)}-{\left\langle \tau_{D\left(A\right)}\right\rangle }_{F}}\right]. \end{array}$$ (27)

This relationship is defined for ${\left\langle \tau_{D\left(A\right)}\right\rangle }_{F}$ in the interval $\left[\tau_{D\left(A\right)}^{\left(1\right)},\tau_{D\left(A\right)}^{\left(2\right)}\right]$, which is equivalent for *x*^(1)^ being in the interval [0, 1]. Because Eq. (27) describes the FRET-line for a binary system in dynamic exchange, we call it the *dynamic FRET-line*. Dynamic FRET-lines connect two static states. They were first introduced by Kalinin *et al.*,^39^ and Gopich and Szabo^73^ later described analogous relations.

**FIG. 4.**
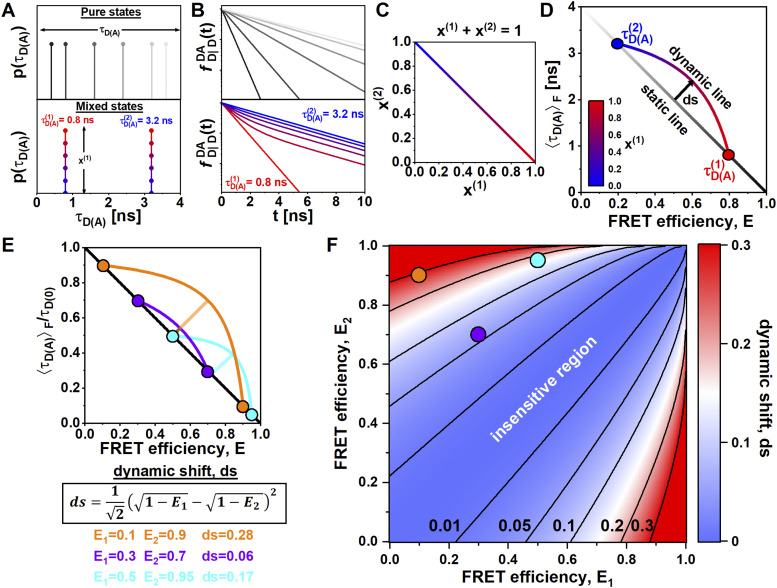
FRET-lines of dynamic molecules. (a) Pure states are characterized by a single lifetime, and the corresponding lifetime distributions show a single peak. In the presence of dynamics, pure states are mixed at different ratios. The lifetime distributions show two peaks weighted by the species fractions ***x***^(1)^ and ***x***^(2)^ = 1 − ***x***^(1)^. The pure states are defined by lifetimes $\tau_{D\left(A\right)}^{\left(1\right)}$ = 0.8 ns and $\tau_{D\left(A\right)}^{\left(2\right)}$ = 3.2 ns. Species fractions are color coded from red ($\left.x^{\left(1\right)}=1\right)$ to blue ($\left.x^{\left(1\right)}=0\right)$. (b) The corresponding fluorescence decays of the lifetime distributions are shown in (a). For pure states, the decays are single exponentials, while mixed states have two-component lifetimes. (c) The dependency between the species fractions ***x***^(1)^ and ***x***^(2)^ is given by ***x***^(1)^ + ***x***^(2)^ = 1. (d) In a plot of the FRET efficiency ***E*** vs the intensity-weighted average fluorescence lifetime ${\left\langle \tau_{D\left(A\right)}\right\rangle }_{F}$, pure states define the static FRET-line (grayscale diagonal line). Mixed states are displaced from the static FRET-line and fall onto a curved line connecting the pure states, described by Eq. (27). The dynamic FRET-line is color-coded by the contribution of species 1. The arrow indicates the maximum possible dynamic shift ds from the static FRET-line. (e) Exemplary dynamic FRET-lines for limiting states with FRET efficiencies *E*_1_ = 0.1/*E*_2_ = 0.9 (orange, ds = 0.28), *E*_1_ = 0.3/*E*_2_ = 0.7 (purple, ds = 0.06), and *E*_1_ = 0.5/*E*_2_ = 0.95 (cyan, ds = 0.17) are shown in a plot of the FRET efficiency vs the normalized intensity-weighted average fluorescence lifetime ${\left\langle \tau_{D\left(A\right)}\right\rangle }_{F}/\tau_{D\left(0\right)}$. (f) Contour plot of the dynamic shift, ds, as a function of the FRET efficiencies of the limiting states, *E*_1_ and *E*_2_. The dynamic shifts for the examples given in *E* are shown as circles. In (d) and (e), the static FRET-lines are given by Eq. (22). Dynamic FRET-lines were calculated according to Eq. (27).

Figure 4 illustrates the concept of static and dynamic FRET-lines. Static FRET-lines describe pure states, which are described by sharp distributions (*δ*-functions) in terms of the lifetime distribution $p\left(\tau_{D\left(A\right)}\right)$ [Fig. 4(a)]. In contrast, dynamic FRET-lines describe the mixing of two pure states as a function of the state occupancy *x*^(1)^. The corresponding donor fluorescence decays are single-exponential for pure states and bi-exponential in the case of mixing between pure states [Fig. 4(b)]. In the $E-{\left\langle \tau_{D\left(A\right)}\right\rangle }_{F}$ plot, the dynamic FRET-line connects the two points of the contributing pure states on the static FRET-line by a curved line [Fig. 4(d)].

To quantify the sensitivity of the dynamic exchange, it is helpful to consider the maximum separation between the dynamic and static FRET-lines. We define this *dynamic shift*, ds, orthogonal to the static FRET-line [Fig. 4(d)]. Like the dynamic FRET-line, the value of the dynamic shift depends only on the FRET efficiencies of the limiting states *E*_1_ and *E*_2_, and is given by (see the supplementary material, Note 2)

$$ds=\frac{1}{\sqrt{2}}{\left(\sqrt{1-E_{1}}-\sqrt{1-E_{2}}\right)}^{2}.$$ (28)

Note that this equation for the dynamic shift is valid for a plot of the FRET efficiency *E* against the *normalized* intensity-averaged donor fluorescence lifetime, ${\left\langle \tau_{D\left(A\right)}\right\rangle }_{F}/\tau_{D\left(0\right)}$. Exemplary dynamic FRET-lines for different FRET efficiencies *E*_1_ and *E*_2_ are shown in Fig. 4(e) with their corresponding dynamic shifts. By visualizing the dynamic shift as a function of the FRET efficiencies of the limiting states [Fig. 4(f)], one can define sensitive and insensitive regions depending on a given detectability threshold for the dynamic shift. This threshold depends on how well the experimental setup is calibrated, the accuracy of the fluorescence lifetime estimation, and the measurement statistics that typical threshold values for the detectability of dynamic shifts are on the order of 0.05 or less, potentially reaching a sensitivity of 0.01 for well-calibrated setups and carefully performed experiments. This places the purple dynamic FRET-line shown in Fig. 4(e) on the border of the insensitive region, while the other two examples with a dynamic shift above 0.1 are clearly in the sensitive region.

### D. General definition of FRET-lines

FRET-lines are idealized relations between the FRET-related experimental observables *E* and ${\left\langle \tau_{D\left(A\right)}\right\rangle }_{F}$ for different physical models of the system. Before considering more specific scenarios, such as the effect of the flexible dye linkers or disordered systems, we first present a general definition of FRET-lines.

Consider that the experiment is described by a physical model defined by a set of parameters Λ. The model encompasses all parameters of the experimental system and fully defines the two-dimensional distribution of the experimental observables, $p\left(E,{\left\langle \tau_{D\left(A\right)}\right\rangle }_{F}|\Lambda \right)$. For a complete description of the experiment, we would require the joint distribution of the experimental observables over the different realizations of the system parameters Λ, weighted by their probability of occurrence *p*(Λ),

$$p\left(E,{\left\langle \tau_{D\left(A\right)}\right\rangle }_{F}\right)=\int p\left(E,{\left\langle \tau_{D\left(A\right)}\right\rangle }_{F}|\Lambda \right)p\left(\Lambda \right)d\Lambda .$$ (29)

This distribution is generally challenging to address as it depends on the photon statistics of the experiment; however, a derivation of the distribution for a two-state system may be found in Ref. 73.

In the ideal case of zero photon shot noise, the distribution $p\left(E,{\left\langle \tau_{D\left(A\right)}\right\rangle }_{F}|\Lambda \right)$ would simplify to ideal curves on the ($E,\left.{\left\langle \tau_{D\left(A\right)}\right\rangle }_{F}\right)$ plane, which define parametric relations between *E* and ${\left\langle \tau_{D\left(A\right)}\right\rangle }_{F}$ as a function of the model parameters Λ. If we choose a fixed value for all model parameters, we obtain a single point on the $\left(E,{\left\langle \tau_{D\left(A\right)}\right\rangle }_{F}\right)$ plane. If, instead, we vary a single parameter, a defined curve—the FRET-line—is obtained. Let the variable parameter be *λ* and the fixed values for the remaining model parameters be Λ_*f*_. Then, the parametric relation between *E* and ${\left\langle \tau_{D\left(A\right)}\right\rangle }_{F}$ for a given model is obtained from the moments of the lifetime distribution by the following equations:

$$E=1-\frac{\bar{\tau_{D\left(A\right)}}\left(\lambda ,\Lambda_{f}\right)}{\tau_{D\left(0\right)}},$$ (30)

$${\left\langle \tau_{D\left(A\right)}\right\rangle }_{F}=\frac{\bar{\tau_{D\left(A\right)}^{2}}\left(\lambda ,\Lambda_{f}\right)}{\bar{\tau_{D\left(A\right)}}\left(\lambda ,\Lambda_{f}\right)}.$$ (31)

To derive the FRET-line for a given physical model, one must compute the moments of the lifetime distribution, $\bar{\tau_{D\left(A\right)}}$ and $\bar{\tau_{D\left(A\right)}^{2}}$, as functions of the model parameters. As an example, our physical model might define the dynamic exchange between two distinct conformations, as described in Sec. III C. In this case, the parameters of the model are the FRET efficiencies of the distinct conformations and the fractional occupancy of the states, i.e., $\Lambda =\{E^{\left(1\right)},E^{\left(2\right)},x^{\left(1\right)},x^{\left(2\right)}\}$, whereby we only have to consider one fractional occupancy as $x^{\left(2\right)}=1-x^{\left(1\right)}$. From this set of parameters, we have chosen *x*^(1)^ as the free parameters ($\left.\lambda =x^{\left(1\right)}\right.$) and kept the FRET efficiencies constant ($\Lambda_{f}=\left\{E^{\left(1\right)},E^{\left(2\right)}\right\}$).

We can write a general expression for the first and second moments of the lifetime in Eqs. (30) and (31) using the definition of the moments,

$$\bar{\tau_{D\left(A\right)}^{\nu }}\left(\lambda ,\Lambda_{f}\right)=\int \tau_{D\left(A\right)}^{\nu }p\left(\tau_{D\left(A\right)}|\lambda ,\Lambda_{f}\right)d\tau_{D\left(A\right)},\nu =\left\{1,2\right\}.$$ (32)

Thus, the problem reduces to find an expression of the lifetime distribution $p\left(\tau_{D\left(A\right)}|\lambda ,\Lambda_{f}\right)$ for a given model. If such an expression is available, we can derive equations for *E* and ${\left\langle \tau_{D\left(A\right)}\right\rangle }_{F}$ (or any related observable) as a function of the variable parameter *λ*. Finally, to obtain the explicit form of the FRET-line, the free parameter *λ* can be eliminated by substitution, and the resulting expression defines a direct relation between the observables *E* and ${\left\langle \tau_{D\left(A\right)}\right\rangle }_{F}$. A detailed description of this general formalism is given in the supplementary material, Note 3.

### E. Experimental observables and moments of the lifetime distribution

The theoretical description of the average delay time $\left\langle t\right\rangle$ and the FRET efficiency in Secs. II C and II D had naturally led us to the moments of the lifetime distribution [Eq. (32)]. The first moment of *p*(*τ*_*D*(*A*)_) is equal to the expected value of the fluorescence lifetime. The second moment is given by the expected value of the square of the fluorescence lifetime. The variance Var(*τ*_*D*(*A*)_) is the second *central* moment, defined as the average squared deviation from the mean, which is related to the first and second moments by

$$\mathrm{Var}\left(\tau_{D\left(A\right)}\right)=\bar{{\left(\tau_{D\left(A\right)}-\bar{\tau_{D\left(A\right)}}\right)}^{2}}=\bar{\tau_{D\left(A\right)}^{2}}-{\bar{\tau_{D\left(A\right)}}}^{2}.$$ (33)

Thus, the second moment and, consequently, ${\left\langle \tau_{D\left(A\right)}\right\rangle }_{F}$ relate to the *variance* of the lifetime distribution. Using the relations between the experimental observables *E* and ${\left\langle \tau_{D\left(A\right)}\right\rangle }_{F}$ and the moments of the lifetime distribution, we obtain

$$\mathrm{Var}\left(\tau_{D\left(A\right)}\right)={\left\langle \tau_{D\left(A\right)}^{2}\right\rangle }_{x}-{{\left\langle \tau_{D\left(A\right)}\right\rangle }_{x}}^{2}=\left(1-E\right)\left(E-E_{\tau }\right)\tau_{D\left(0\right)}^{2},$$ (34)

where we have introduced the quantity *E*_*τ*_ defined as

$$E_{\tau }=1-\frac{{\left\langle \tau_{D\left(A\right)}\right\rangle }_{F}}{\tau_{D\left(0\right)}}.$$ (35)

Note that we have used different notations for the expected moments of the lifetime distribution as defined by Eq. (32), denoted as $\bar{\tau_{D\left(A\right)}^{\nu }}$, compared to the estimates of these moments derived from the experimental observables *E* and ${\left\langle \tau_{D\left(A\right)}\right\rangle }_{F}$, denoted as ${\left\langle \tau_{D\left(A\right)}^{\nu }\right\rangle }_{x}$, to make a clear distinction between the expected theoretical quantities and the experimental estimates.

Due to the linear relation between the FRET efficiency and the fluorescence lifetime, the variance of the lifetime distribution is directly proportional to the variance of the FRET efficiency distribution by

$$\mathrm{Var}\left(E\right)=\frac{\mathrm{Var}\left(\tau_{D\left(A\right)}\right)}{\tau_{D\left(0\right)}^{2}}.$$ (36)

This provides an alternate approach to BVA to estimate the variance of the FRET efficiency distribution from the observables *E* and ${\left\langle \tau_{D\left(A\right)}\right\rangle }_{F}$. The result is identical to the expression obtained in Ref. 73, relating ${\left\langle \tau_{D\left(A\right)}\right\rangle }_{F}$ to the variance of the FRET efficiency distribution,

$${\left\langle \tau_{D\left(A\right)}\right\rangle }_{F}=\tau_{D\left(0\right)}\left[1-E+\frac{\mathrm{Var}\left(E\right)}{1-E}\right].$$ (37)

For a single lifetime component, the distribution of lifetimes is given by a Dirac delta function *δ*,

$$p\left(\tau_{D\left(A\right)}\right)=x^{\left(i\right)}\delta \left(\tau -\tau_{D\left(A\right)}^{\left(i\right)}\right)=\left\{\begin{array}{cc} x^{\left(i\right)},\; & \tau_{D\left(A\right)}=\tau_{D\left(A\right)}^{\left(i\right)},\\ 0\; & \text{else}. \end{array}\right.$$ (38)

The *v*th moment is then given by $\tau_{D\left(A\right)}^{\nu }$, and the variance of the distribution, as given by Eq. (33), is zero, defining the equivalent static FRET-line. Thus, the static FRET-line [Eq. (22)] corresponds to the particular case of lifetime distributions with vanishing variance. For two-component lifetime distributions, the distribution of lifetimes is given by the weighted sum of two *δ*-functions, leading to the following expression for the moments of the lifetime distribution:

$$\begin{array}{c} {\left\langle \tau_{D\left(A\right)}\right\rangle }_{x}=x^{\left(1\right)}\tau_{D\left(A\right)}^{\left(1\right)}+\left(1-x^{\left(1\right)}\right)\tau_{D\left(A\right)}^{\left(2\right)},\\ {\left\langle \tau_{D\left(A\right)}^{2}\right\rangle }_{x}=x^{\left(1\right)}{\left(\tau_{D\left(A\right)}^{\left(1\right)}\right)}^{2}+\left(1-x^{\left(1\right)}\right){\left(\tau_{D\left(A\right)}^{\left(2\right)}\right)}^{2}. \end{array}$$ (39)

Note that the moments of the lifetime distribution are linear functions of the species fraction *x*^(1)^. For the mixing between two states [Eq. (39)], the variance is then given by

$$\mathrm{Var}\left(\tau_{D\left(A\right)}\right)=\bar{\tau_{D\left(A\right)}^{2}}-{\bar{\tau_{D\left(A\right)}}}^{2}=x^{\left(1\right)}\left(1-x^{\left(1\right)}\right){\left(\tau_{D\left(A\right)}^{\left(1\right)}-\tau_{D\left(A\right)}^{\left(2\right)}\right)}^{2}.$$ (40)

We can eliminate the variable *x*^(1)^ to obtain the relation between $\mathrm{Var}\left(\tau_{D\left(A\right)}\right)$ and ${\left\langle \tau_{D\left(A\right)}\right\rangle }_{x}$,

$$\mathrm{Var}\left(\tau_{D\left(A\right)}\right)=\left[{\left\langle \tau_{D\left(A\right)}\right\rangle }_{x}-\tau_{D\left(A\right)}^{\left(1\right)}\right]\left[\tau_{D\left(A\right)}^{\left(2\right)}-{\left\langle \tau_{D\left(A\right)}\right\rangle }_{x}\right],$$ (41)

from which the variance of the FRET efficiency distribution is obtained by Eq. (36). Equation (41) defines the dynamic FRET-line for data displayed in the mean-variance representation.

**FIG. 5.**
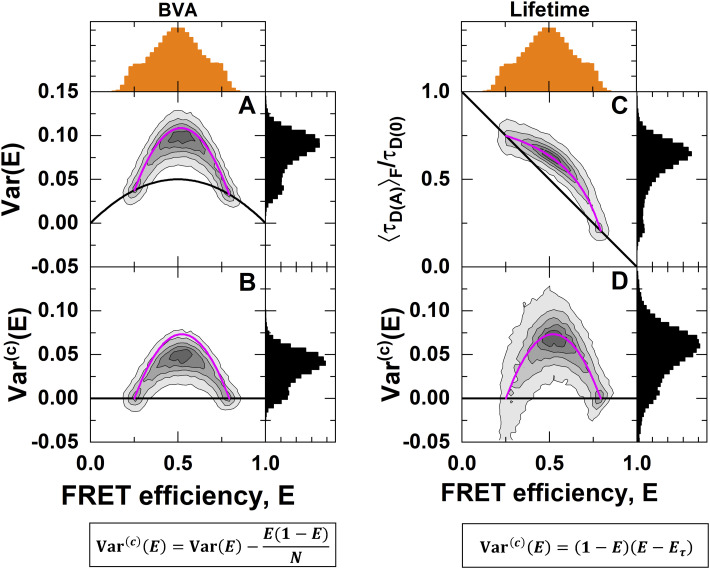
Estimating the variance of the FRET efficiency distribution. Shown is a simulation of molecules interconverting between two distinct states with ***E***^(1)^ = 0.25 and ***E***^(2)^ = 0.8, interconversion rates of ***k***_12_ = ***k***_21_ = 1 ms^−1^, and a diffusion time of 0.5 ms. (a) Burst variance analysis (BVA) quantifies the total variance of the FRET efficiency through analysis of the photon time trace [compare Fig. 3(a)], which contains contributions from photon shot noise and conformational dynamics (magenta line). The shot noise variance is given as a black line. The static FRET-line (black) is given by Eq. (21), and the dynamic FRET-line (magenta) is given by the sum of the variance caused by conformational dynamics, given by Eqs. (36) and (41), and shot noise as given by Eq. (21). (b) A simple subtraction of the photon shot noise reveals the variance due to conformational dynamics, $Var^{\left(c\right)}\left(E\right)$. The dynamic FRET-line is given by Eqs. (36) and (41). (c) The plot of the two observables ***E*** and ${\left\langle \tau_{D\left(A\right)}\right\rangle }_{F}$ reveals the dynamics as a right-ward shift from the static FRET-line (black). The static FRET-line is given by Eq. (22), and the dynamic FRET-line (magenta) was calculated according to Eq. (27). (d) The estimated variance of the FRET efficiency from the observables follows the expected line as given by Eq. (41).

To illustrate that, we can indeed estimate the variance of the FRET efficiency distribution from the two experimental observables *E* and ${\left\langle \tau_{D\left(A\right)}\right\rangle }_{F}$, we compare the variance estimate with that obtained from burst variance analysis (BVA) for a simulated dataset (Fig. 5).

BVA correctly identifies the presence of conformational dynamics between the two states at FRET efficiencies of 0.25 and 0.8 [Fig. 5(a)]. The variance estimate obtained from BVA, however, includes the contribution of photon shot noise Eq. (21) [black line in Fig. 5(a)], and the dynamics is shown as excess variance beyond the shot noise limit.

**FIG. 6.**
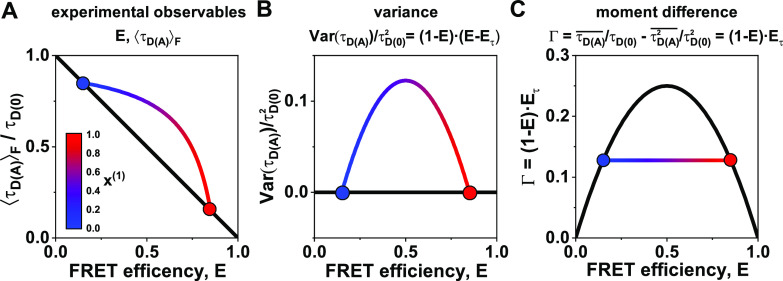
Different representations of the FRET estimators ***E*** and ${\left\langle \tau_{D\left(A\right)}\right\rangle }_{F}$ and the derived quantities for a two-state system with ***E***^(1)^ = 0.25 and ***E***^(2)^ = 0.75. (a) In a plot of the two observables ***E*** and ${\left\langle \tau_{D\left(A\right)}\right\rangle }_{F}$, dynamics show as a curved line (color-coded by the contribution of the low-FRET species) that deviates from the diagonal static FRET-line (black). The static FRET-line is given by Eq. (21), and the dynamic FRET-line (gradient line) was calculated according to Eq. (27). (b) In the mean-variance representation (bottom), the static FRET-line is given by zero variance, while the dynamic line curves upward. The dynamic FRET-line (gradient line) was calculated according to Eq. (41). (c) Using the difference between the first and second moments **Γ**, the static FRET-line transforms into a parabola, while the dynamic FRET-line is given by a line. All lifetimes and moments are normalized to the donor-only lifetime $\tau_{D\left(0\right)}$ to simplify the illustration. The static FRET line (black) is given by Eq. (45), and the dynamic FRET-line (gradient line) was calculated according to Eq. (47).

To obtain the contribution to the variance due to conformational dynamics ($Var^{\left(c\right)}\left(E\right)$), we subtract the shot noise contribution given by $\sigma_{SN}^{2}=E\left(1-E\right)/N$, where *N* = 5 is the photon window used for the analysis [Fig. 5(b)]. Compared to the expected variance given by Eq. (41) (pink line), BVA underestimates the variance of the FRET efficiency caused by the averaging over the photon window used in the calculation. It must also be considered that BVA measures the combined variance of the FRET efficiency caused by the contributions of shot noise and dynamics. However, these contributions are not strictly additive. The simple subtraction of the shot noise contribution performed here is, thus, only approximative. In the (*E*, ${\left\langle \tau_{D\left(A\right)}\right\rangle }_{F}$) representation, the same dataset shows a dynamic shift from the diagonal line that is described by the dynamic FRET-line [Fig. 5(c)]. From the experimental observables, we calculate the variance of the FRET efficiency distribution. Unlike the variance obtained by BVA, this variance estimate represents the pure contribution of the conformational dynamics and follows the expected dynamic FRET-line. Note, however, that the molecule-wise distribution of the variance estimated from the observables *E* and ${\left\langle \tau_{D\left(A\right)}\right\rangle }_{F}$ generally shows a broader distribution compared to BVA. Conceptual static and two-state dynamic FRET-lines for the mean-variance representation of the data are shown in Fig. 6(b).

### F. Alternative representation of dynamic lines

For the dynamic mixing between pure species, i.e., species whose lifetime distributions are described by *δ*-functions, the moments of the lifetime distribution are simply given by the linear combination of the moments of the pure components [compare Eq. (39)],

$$p^{\left(i\right)}\left(\tau_{D\left(A\right)}\right)=\sum x^{\left(i\right)}\delta \left(\tau -\overset{\left(i\right)}{\underset{D\left(A\right)}{\tau }}\right)\Rightarrow \begin{array}{c} {\left\langle \tau_{D\left(A\right)}\right\rangle }_{x}=\sum x^{\left(i\right)}\overset{\left(i\right)}{\underset{D\left(A\right)}{\tau }},\\ {\left\langle \tau_{D\left(A\right)}^{2}\right\rangle }_{x}=\sum x^{\left(i\right)}{\left(\overset{\left(i\right)}{\underset{D\left(A\right)}{\tau }}\right)}^{2}. \end{array}$$ (42)

Any linear combination of the quantities ${\left\langle \tau_{D\left(A\right)}\right\rangle }_{x}$ and ${\left\langle \tau_{D\left(A\right)}^{2}\right\rangle }_{x}$ will, thus, retain this property. This implies that we can further simplify the expression for the dynamic FRET-line by choosing the first and second moments as the parameters, which results in a linear expression for the dynamic FRET-line.

In the parameter space of the first two moments $\left({\left\langle \tau_{D\left(A\right)}\right\rangle }_{x}\right.$, $\left.{\left\langle \tau_{D\left(A\right)}^{2}\right\rangle }_{x}\right)$, the static FRET-line is given by ${\left\langle \tau_{D\left(A\right)}^{2}\right\rangle }_{x}={\left({\left\langle \tau_{D\left(A\right)}\right\rangle }_{x}\right)}^{2}$, which is the equation for an ordinary parabola. In other words, while the dynamic FRET-line is linearized, we now have a quadratic relation for the static FRET-line. Using the parameters (${\left\langle \tau_{D\left(A\right)}\right\rangle }_{x}$, ${\left\langle \tau_{D\left(A\right)}^{2}\right\rangle }_{x}$), static and dynamic FRET-lines are, however, not well separated, making it challenging to distinguish static from dynamic molecules. To overcome this problem, we replace the second moment with the difference between the normalized first and second moments,

$$\Gamma =\frac{{\left\langle \tau_{D\left(A\right)}\right\rangle }_{x}}{\tau_{D\left(0\right)}}-\frac{{\left\langle \tau_{D\left(A\right)}^{2}\right\rangle }_{x}}{\tau_{D\left(0\right)}^{2}}.$$ (43)

This *moment difference* Γ is related to the experimental observables *E* and ${\left\langle \tau_{D\left(A\right)}\right\rangle }_{F}$ by

$$\Gamma =\left(1-E\right)\left(1-\frac{{\left\langle \tau_{D\left(A\right)}\right\rangle }_{F}}{\tau_{D\left(0\right)}}\right)=\left(1-E\right)E_{\tau },$$ (44)

where we defined $E_{\tau }=1-\frac{{\left\langle \tau_{D\left(A\right)}\right\rangle }_{F}}{\tau_{D\left(0\right)}}$.

In this representation, the static FRET-line transforms to

$$\begin{array}{ll} \Gamma_{static} & =\frac{{\left\langle \tau_{D\left(A\right)}\right\rangle }_{x}}{\tau_{D\left(0\right)}}-{\frac{\left({\left\langle \tau_{D\left(A\right)}\right\rangle }_{x}\right)}{\tau_{D\left(0\right)}^{2}}}^{2}\\ & =\frac{{\left\langle \tau_{D\left(A\right)}\right\rangle }_{x}}{\tau_{D\left(0\right)}}\left(1-\frac{{\left\langle \tau_{D\left(A\right)}\right\rangle }_{x}}{\tau_{D\left(0\right)}}\right)=\left(1-E\right)E. \end{array}$$ (45)

Equation (45) describes a parabola that crosses the FRET efficiency axis at points (0, 0) and (1, 0) and has a maximum at (1/2, 1/4) [Fig. 6(c)]. In the case of dynamics, the difference of the normalized lifetime moments is given by

$$\begin{array}{ll} \Gamma_{dynamic} & =x^{\left(1\right)}\frac{\tau^{\left(1\right)}}{\tau_{D\left(0\right)}}\left(1-\frac{\tau^{\left(1\right)}}{\tau_{D\left(0\right)}}\right)+\left(1-x^{\left(1\right)}\right)\frac{\tau^{\left(2\right)}}{\tau_{D\left(0\right)}}\left(1-\frac{\tau^{\left(2\right)}}{\tau_{D\left(0\right)}}\right). \end{array}$$ (46)

From this, we obtain the simple form of the dynamic FRET-line,

$$\begin{array}{ll} \Gamma_{dynamic} & =\left(1-\frac{\tau^{\left(1\right)}}{\tau_{D\left(0\right)}}-\frac{\tau^{\left(2\right)}}{\tau_{D\left(0\right)}}\right)\frac{{\left\langle \tau_{D\left(A\right)}\right\rangle }_{x}}{\tau_{D\left(0\right)}}+\frac{\tau^{\left(1\right)}\tau^{\left(2\right)}}{\tau_{D\left(0\right)}^{2}}\\ & =\left(1-E^{\left(1\right)}-E^{\left(2\right)}\right)E+E^{\left(1\right)}E^{\left(2\right)}. \end{array}$$ (47)

The expression for the dynamic FRET-line is linear to the FRET efficiency *E*, directly connecting the two points belonging to the pure states [Fig. 6(c)].

In the difference between the first and second normalized moments Γ, we found a parameter that linearizes the dynamic mixing while retaining a simple relation for the static FRET-line. The linearization of dynamics in this *moment representation* dramatically simplifies the graphical analysis of kinetic networks by providing a direct visualization of the kinetic connectivity. To highlight its usefulness, we show the moment representation together with the histogram of the experimental observables, *E* and ${\left\langle \tau_{D\left(A\right)}\right\rangle }_{F}$, in the following discussions of more complex scenarios. The moment representation resembles the analysis of fluorescence lifetimes in the phasor approach to fluorescence lifetime imaging (Phasor-FLIM).^84^ In both approaches, single-exponential fluorescence decays are found on a curve, a parabola in the moment representation, and a circle in Phasor-FLIM. Moreover, bi-exponential decays are shifted inward from the curve and lie on the line connecting the coordinates of the pure components. The phasor calculation only requires fluorescence decays and, thus, is also applicable to study quenching without FRET. In principle, the moment representation could thus be combined with the phasor information to add another dimension to the analysis. The different transformations of the observables *E* and ${\left\langle \tau_{D\left(A\right)}\right\rangle }_{F}$ and their theoretical equivalents are summarized in Table I.

**TABLE I.** Overview of the experimental parameters and the corresponding model parameters.

Model		Experiment
Probability distribution	↔	Random realization
Expected value	↔	Experimental observable
Probability density function	↔	Histogram
FRET-lines	↔	Distribution of FRET efficiency, fluorescence lifetime, or related quantities
Expectation value of FRET efficiency	↔	Species-averaged FRET efficiency
$E=1-\frac{\bar{\tau_{D\left(A\right)}}}{\tau_{D\left(0\right)}}$	$\overset{\wedge }{=}$	$E=\frac{F_{A|D}}{F_{A|D}+F_{D|D}}=1-\frac{{\left\langle \tau_{D\left(A\right)}\right\rangle }_{x}}{\tau_{D\left(0\right)}}$
First moment of the lifetime distribution	↔	Species-averaged lifetime
$\bar{\tau_{D\left(A\right)}}=\overset{\infty }{\underset{0}{\int }}\tau_{D\left(A\right)}p\left(\tau_{D\left(A\right)}\right)d\tau_{D\left(A\right)}$	$\overset{\wedge }{=}$	${\left\langle \tau_{D\left(A\right)}\right\rangle }_{x}=\left(1-E\right)\tau_{D\left(0\right)}$
Second moment of the lifetime distribution	↔	Species-averaged *squared* lifetime
$\bar{\tau_{D\left(A\right)}^{2}}=\overset{\infty }{\underset{0}{\int }}\tau_{D\left(A\right)}^{2}p\left(\tau_{D\left(A\right)}\right)d\tau_{D\left(A\right)}$	$\overset{\wedge }{=}$	${\left\langle \tau_{D\left(A\right)}^{2}\right\rangle }_{x}={\left\langle \tau_{D\left(A\right)}\right\rangle }_{F}\left(1-E\right)\tau_{D\left(0\right)}$
Ratio of the second and first moments of the	↔	Intensity-weighted average fluorescence lifetime, average delay time
lifetime distribution				
$\frac{\bar{\tau_{D\left(A\right)}^{2}}}{\bar{\tau_{D\left(A\right)}}}$	$\overset{\wedge }{=}$	${\left\langle \tau_{D\left(A\right)}\right\rangle }_{F}\;\mathrm{or}\;\left\langle t\right\rangle$
No equivalent	↔	Intensity-weighted average FRET efficiency
$1-\frac{1}{\tau_{D\left(0\right)}}\frac{\bar{\tau_{D\left(A\right)}^{2}}}{\bar{\tau_{D\left(A\right)}}}$	$\overset{\wedge }{=}$	$E_{\tau }=1-\frac{{\left\langle \tau_{D\left(A\right)}\right\rangle }_{F}}{\tau_{D\left(0\right)}}$
Variance of the lifetime distribution	↔	No equivalent
$\mathrm{Var}\left(\tau_{D\left(A\right)}\right)=\bar{\tau_{D\left(A\right)}^{2}}-{\bar{\tau_{D\left(A\right)}}}^{2}=\mathrm{Var}\left(E\right)\tau_{D\left(0\right)}^{2}$	$\overset{\wedge }{=}$	$\left(1-E\right)\left(E-E_{\tau }\right)\tau_{D\left(0\right)}^{2}$
Difference between the normalized first and second	↔	No equivalent
moments of the lifetime distribution				
$\Gamma =\frac{\bar{\tau_{D\left(A\right)}}}{\tau_{D\left(0\right)}}-\frac{\bar{\tau_{D\left(A\right)}^{2}}}{{\tau_{D\left(0\right)}}^{2}}$	$\overset{\wedge }{=}$	(1 − *E*)*E*_*τ*_

### G. Multi-state systems

**FIG. 7.**
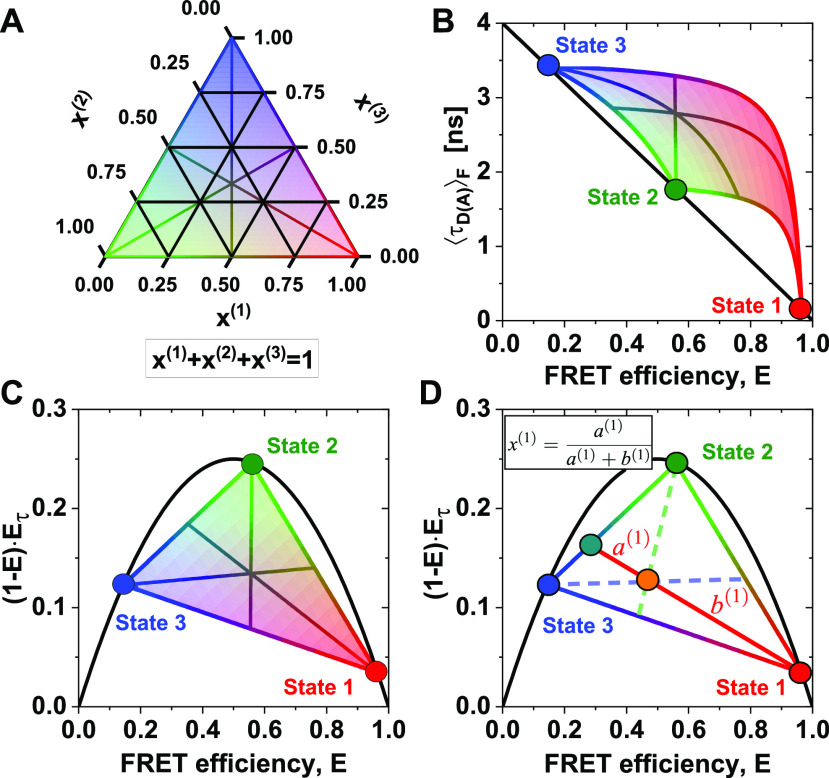
FRET-lines in three-state systems. (a) Ternary plot of the fractions of the three species. The area is colored according to the contribution of the species (red: ***x***^(1)^, green: ***x***^(2)^, blue: ***x***^(3)^). (b) and (c) In the $\left(E,{\left\langle \tau_{D\left(A\right)}\right\rangle }_{F}\right.$) parameter space (b), three-state mixing is described by an area that is confined by the two-state dynamic FRET-lines. In the moment representation (c), the dynamic mixing is simplified to a triangle with straight lines that describe the dynamic interconversion. Additionally, specific examples of limiting FRET-lines are given in (a)–(c). For these lines, one species fraction is varied, while the other two fractions are kept equal, e.g., $x^{\left(1\right)}\in \left[0,1\right]$ and $x^{\left(2\right)}=x^{\left(3\right)}=0.5\left(1-x^{\left(1\right)}\right)$. These lines intersect at ***x***^(1)^ = ***x***^(2)^ = ***x***^(3)^ = 1/3. In (b), the static FRET-line (black) is given by Eq. (21) and the limiting dynamic FRET-lines were calculated according to Eq. (27). In (c), the static FRET-line (black) is given by Eq. (45) and the limiting dynamic FRET-lines were calculated according to Eq. (47). (d) In the moment representation, the species fractions can be determined by graphical analysis from the sections ***a***^(1)^ and ***b***^(1)^ of the connecting line between the position of the population (orange) and the pure states. The solid line indicates the procedure to determine the fraction of species 1, while the corresponding lines for species 2 and 3 are given as dashed lines.

The concept of FRET-lines is beneficial to characterize complex kinetic schemes with more than two states. Consider a kinetic network involving three conformational states,
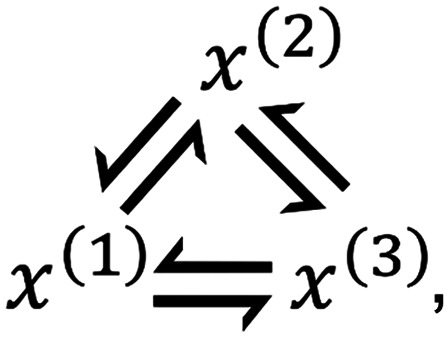
(48)with the fraction of the three states $x^{\left(1\right)}$, $x^{\left(2\right)}$, and $x^{\left(3\right)}$, where $x^{\left(3\right)}=1-x^{\left(1\right)}-x^{\left(2\right)}$. The equations for the moments of the lifetime distribution are easily extended for the three-state system, but we can only eliminate one of the two free parameters $x^{\left(1\right)}$ and $x^{\left(2\right)}$. Consequently, the conversion function between species-averaged and fluorescence-averaged fluorescence lifetime additionally depends on one of the three species fractions, and we can only define the equivalent of FRET-lines by fixing this species fraction at a specific value. Because there are, thus, two degrees of freedom, multi-state systems are described by an area instead of a line [Figs. 7(a)–7(c)]. This area is enclosed by limiting binary dynamic FRET-lines, which describe the direct exchange among two of the three states and are obtained by fixing one of the species fractions to zero. To define multi-state FRET-lines analogous to the two-state system, it is necessary to include an additional boundary condition. For example, the lines crossing the area in Figs. 7(a)–7(c) are obtained by varying one of the fractions while requiring the other two to be equal.

When more than two states are involved, the equilibrium population potentially lies enclosed by the limiting binary dynamic FRET-lines. The dynamic FRET area then serves as a reference to reveal the spatial and temporal heterogeneities of the sample. The color of the area in Figs. 7(a)–7(c) represents the population fraction of each of the states. The position of a single-molecule event on the plane is related to the occupancy fractions *x*^(*i*)^ of the measured molecule, which in the limit of fast dynamics (or long observation time) tend to the equilibrium fractions. Using the representation of the moment difference [Fig. 7(c)], it is possible to determine the state occupancies of the different states graphically from the two-dimensional plot [Fig. 7(d)]. As an example, we consider the high-FRET state 1 [red circle in Fig. 7(d)]. The red line connecting state 1 to the mixed population (orange) intersects the binary exchange line between states 2 (green) and 3 (blue) at a given point (turquoise). Then, the state occupancy *x*^(1)^ is obtained from the length of the segments of the red line, *a*^(1)^ and *b*^(1)^, defined by the position of the mixed population along the line, by

$$x^{\left(1\right)}=\frac{a^{\left(1\right)}}{a^{\left(1\right)}+b^{\left(1\right)}}.$$ (49)

The state occupancies *x*^(2)^ and *x*^(3)^ are obtained analogously as described for *x*^(1)^ above, as indicated by the dashed lines in Fig. 7(c). A detailed derivation of this expression is given in the supplementary material, Note 4.

To draw such multi-state FRET-lines, it is important that the parameters of the limiting states are known. How easily this information is gathered depends on the system at hand. For slow dynamics, residual populations of pseudo-static molecules, i.e., molecules that by chance did not convert during the observation time, clearly indicate the location of the limiting states in the two-dimensional plots. On the other hand, in the case of fast dynamics, only a single population might be observed, which deviates from the static FRET-line. In this case, the fluorescence lifetimes of the limiting states might be identified from a sub-ensemble TCSPC analysis. However, distinguishing between different numbers of states in such an analysis is challenging if a low number of photons is detected, and it is recommended that a network of FRET pairs is globally analyzed.^85^ Often, the conformational equilibrium can also be modulated, allowing the populations to be shifted toward or locked into a certain conformation. Finally, prior information on the structure of stable conformational states, e.g., obtained from x-ray crystallography or nuclear magnetic resonance (NMR) spectroscopy, can be used to predict the parameters of the limiting states.

In multi-state systems, FRET-lines are especially helpful in identifying the minimal set of states and their kinetic connectivity. This information can reduce the complexity of the kinetic model by eliminating exchange pathways, providing crucial information for further quantitative analysis of the dynamic network by dynamic photon distribution analysis or fluorescence correlation spectroscopy. This aspect of FRET-lines is illustrated in detail in Paper II.

## IV. PRACTICAL ASPECTS AND APPLICATION

### A. Multi-exponential donor decays

Up until now, we have assumed that the fluorescence decay of the donor dye in the absence of the acceptor is single exponential. Experimentally, however, this condition is often violated due to the effect of the local environment on the tethered dyes. The most common mechanisms that affect the quantum yield of tethered dyes are the quenching of rhodamine or xanthene based dyes by electron-rich amino acids, such as tryptophane, through photo-induced electron transfer (PET)^86–88^ and the enhancement of the fluorescence of cyanine-based dyes due to steric restriction and dye–surface interactions that modulate the cis–trans isomerization.^89–91^ In addition, the used organic dyes may consist of a mixture of isomers with distinct fluorescence properties. The effect of multi-exponential fluorescence decays of the donor fluorophore on the static and dynamic FRET-lines depends on the timescale of the dynamic exchange between the different donor states. This exchange may be fast (e.g., in the case of dynamic quenching by PET), on a similar timescale as the observation time of a few milliseconds (e.g., for sticking of the fluorophore to the biomolecular surface), or non-existent (e.g., in the case of an isomer mixture).

Here, we consider two limiting cases of donor dyes with multi-exponential fluorescence decays in the absence of FRET: a static mixture and fast exchange with complete averaging during the observation time. As before, we assume the homogeneous approximation wherein the fluorescence properties are identical in different conformational states of the host molecule, i.e., the FRET rate *k*_*RET*_ does not depend on the donor-only lifetime *τ*_*D*(0)_. In this case, the donor fluorescence decay in the absence of FRET is described by a distribution of fluorescence lifetimes *p*(*τ*_*D*(0)_),

$$f_{D|D}^{\left(D0\right)}\left(t\right)=k_{F,D}\int p\left(\tau_{D\left(0\right)}\right)e^{-t/\tau_{D\left(0\right)}}d\tau_{D\left(0\right)}.$$ (50)

For the donor fluorescence decay in the presence of the acceptor, we now have to consider a distribution of donor fluorescence lifetimes and FRET rates,

$$f_{D|D}^{\left(DA\right)}\left(t\right)=k_{F,D}\int \int p\left(\tau_{D\left(0\right)}\right)p\left(k_{RET}\right)e^{-t/\tau_{D\left(A\right)}}dk_{RET}d\tau_{D\left(0\right)},$$ (51)

where the donor fluorescence lifetime in the presence of the acceptor is given by $\tau_{D\left(A\right)}={\left(\tau_{D\left(0\right)}^{-1}+k_{RET}\right)}^{-1}$, and *p*(*τ*_*D*(0)_) and $p\left(k_{RET}\right)$ correspond to the donor-only lifetimes and FRET rate distributions, respectively. Note that due to the homogeneous approximation, we have factored the joint distribution of donor and FRET states, that is, $p\left(\tau_{D\left(0\right)},k_{RET}\right)=p\left(\tau_{D\left(0\right)}\right)p\left(k_{RET}\right)$.

The moments of the fluorescence lifetime distribution then evaluate to

$$\bar{\tau_{D\left(A\right)}^{\nu }}=\overset{\infty }{\underset{0}{\int }}\overset{\infty }{\underset{0}{\int }}p\left(\tau_{D\left(0\right)}\right)p\left(k_{RET}\right)\tau_{D\left(A\right)}^{\nu }dk_{RET}d\tau_{D\left(0\right)},$$ (52)

or in the discrete case of distinct donor-only and FRET states,

$${\left\langle \tau_{D\left(A\right)}^{\nu }\right\rangle }_{x}=\underset{i,j}{\sum }x_{D\left(0\right)}^{\left(j\right)}x_{RET}^{\left(i\right)}{\left(\tau_{D\left(A\right)}^{\left(i,j\right)}\right)}^{\nu },$$ (53)

where $x_{D\left(0\right)}^{\left(j\right)}$ and $x_{RET}^{\left(i\right)}$ are the fractions of the donor (*j*) and FRET (*i*) states, respectively, and $\tau_{D\left(A\right)}^{\left(i,j\right)}={\left({\left(\tau_{D\left(0\right)}^{\left(j\right)}\right)}^{-1}+k_{RET}^{\left(i\right)}\right)}^{-1}$. From the moments, the observable ${\left\langle \tau_{D\left(A\right)}\right\rangle }_{F}$ is then readily calculated according to Eq. (17).

A more complex situation arises for the intensity-based FRET efficiency *E* because the fluorescence intensities obtained for the different donor states are weighted by their respective quantum yields. Consequently, it becomes impossible to define a single distance-related FRET efficiency. Instead, we define the proximity ratio *E*_*PR*_ in analogy to Eq. (9) based on the average fluorescence intensities detected in the donor and acceptor channels $\bar{F_{D|D}^{\left(DA\right)}}$ and $\bar{F_{A|D}^{\left(DA\right)}}$ by

$$E_{PR}=\frac{\bar{F_{A|D}^{\left(DA\right)}}}{\bar{F_{D|D}^{\left(DA\right)}}+\bar{F_{A|D}^{\left(DA\right)}}}=1-\frac{{\left\langle \tau_{D\left(A\right)}\right\rangle }_{x}}{{\left\langle {\tau^{\prime }}_{D\left(0\right)}\right\rangle }_{x}},$$ (54)

where the species-averaged lifetimes are calculated over all donor and FRET states. The effective donor-only lifetime $\tau_{D\left(0\right)}^{\prime }$ in the presence of quenching is defined as

$$\tau_{D\left(0\right)}^{\prime }=\tau_{D\left(A\right)}+\gamma^{\prime }\left(\tau_{D\left(0\right)}-\tau_{D\left(A\right)}\right),$$ (55)

where the factor *γ*′ is given by the ratio of the quantum yields of the acceptor and donor fluorophores, $\gamma^{\prime }=\frac{\Phi_{F,A}}{\Phi_{F,D}}$. See the supplementary material, Note 5, for a derivation of Eq. (55). For the moment representation, the moment difference Γ in the case of a mixture of donor states is then defined as

$$\Gamma =\left(1-E_{PR}\right)\left(1-\frac{{\left\langle \tau_{D\left(A\right)}\right\rangle }_{F}}{{\left\langle \tau_{D\left(0\right)}\right\rangle }_{F}}\right)=\left(1-E_{PR}\right)E_{PR,\tau },$$ (56)

where ${\left\langle \tau_{D\left(0\right)}\right\rangle }_{F}$ is the intensity-weighted average donor fluorescence lifetime and $E_{PR,\tau }=1-\frac{{\left\langle \tau_{D\left(A\right)}\right\rangle }_{F}}{{\left\langle \tau_{D\left(0\right)}\right\rangle }_{F}}$.

**FIG. 8.**
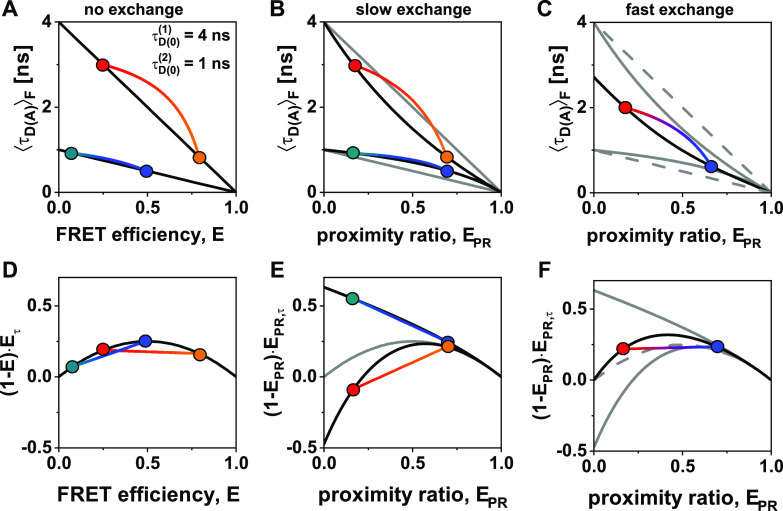
Static and dynamic FRET-lines for mixtures of distinct photophysical states of the donor in the (*E*-${\left\langle \tau_{D\left(A\right)}\right\rangle }_{F}$) parameter space [(a)–(c)] and in the moment representation [(d)–(f)]. (a) and (d) Static and binary dynamic FRET lines for a superposition of two species with distinct donor-only lifetimes of $\tau_{D\left(0\right)}^{\left(1\right)}$ = 4 ns and $\tau_{D\left(0\right)}^{\left(2\right)}$ = 1 ns, corresponding to donor quantum yields of $\Phi_{F,D}^{\left(1\right)}$ = 0.8 and $\Phi_{F,D}^{\left(2\right)}$ = 0.2. Static FRET-lines are shown in black. The inter-dye distances of the two FRET species are $R_{DA}^{\left(1\right)}$ = 40 Å (blue, orange) and $R_{DA}^{\left(2\right)}$ = 60 Å (teal, red). The Förster radius of the donor state with $\tau_{D\left(0\right)}^{\left(1\right)}$ = 4 ns is *R*_0_ = 50 Å. The acceptor quantum yield is chosen as Φ_*F*,*A*_ = 0.8. In (a), the static FRET-lines are given by Eq. (22) and dynamic FRET lines were calculated according to Eq. (27). In (d), the static FRET-line is given by Eq. (45) and the limiting dynamic FRET-lines were calculated according to Eq. (47). (b) and (e) Static and binary dynamic FRET lines for the mixture of the two species shown in (a) and (d) in slow exchange, i.e., on a timescale slower than the observation time, with equilibrium fractions of $x_{D\left(0\right)}^{\left(1\right)}$ = 0.25 and $x_{D\left(0\right)}^{\left(2\right)}$ = 0.75. For the proximity ratio *E*_*PR*_, a curvature of the static FRET-lines arises even in the absence of dynamics. Gray lines correspond to the ideal static FRET-lines shown in (a). Note that in (e), the moment difference $\left(1-E_{PR}\right)E_{PR,\tau }$ can assume negative values. (c) and (f) Static and binary dynamic FRET-lines for the mixture of the two species shown in (a) in fast exchange, i.e., for complete averaging during the observation time, with equilibrium fractions of $x_{D\left(0\right)}^{\left(1\right)}$ = 0.25 and $x_{D\left(0\right)}^{\left(2\right)}$ = 0.75. Solid gray lines correspond to the static FRET-lines for slow exchange as shown in (b) and (e). Dashed gray lines correspond to the ideal static FRET-lines of the two species as shown in (a) and (d). Note that the static FRET-line is convex in this case. In (b) and (c) and (e) and (f), the FRET lines are calculated from the averaged observables defined in Eqs. (53)–(55), assuming a single donor photophysical state for each line.

The effect of a mixture of two distinct photophysical states of the donor is illustrated in Fig. 8 for the (*E*-${\left\langle \tau_{D\left(A\right)}\right\rangle }_{F}$) parameter space [(a)–(c)] and in the moment representation [(d)–(f)]. We consider two different donor lifetimes of $\tau_{D\left(0\right)}^{\left(1\right)}$ = 4 ns and $\tau_{D\left(0\right)}^{\left(2\right)}$ = 1 ns that correspond to distinct donor quantum yields of $\Phi_{F,D}^{\left(1\right)}$ = 0.8 and $\Phi_{F,D}^{\left(2\right)}$ = 0.2. When separate measurements are performed [Figs. 8(a) and 8(d)], accurate FRET efficiencies *E* can be calculated for each measurement, and the ideal static and dynamic FRET-lines are obtained. For the dynamic exchange, we assume equilibrium fractions of $x_{D\left(0\right)}^{\left(1\right)}$ = 0.25 and $x_{D\left(0\right)}^{\left(2\right)}$ = 0.75 for the two donor states. In the case of exchange on a timescale much slower than the observation time [Figs. 8(b) and 8(e)], an individual correction of the different populations is not possible. For the proximity ratio *E*_*PR*_, curved static FRET-lines are obtained for the two species as an effect of the averaging in Eq. (54). In the moment representation, this effect shows as an increased (for the species with $\tau_{D\left(0\right)}^{\left(2\right)}$ = 4 ns) or decreased (for the species with $\tau_{D\left(0\right)}^{\left(2\right)}$ = 1 ns) curvature of the static FRET-lines, while the linearity of the dynamic FRET-lines is retained. The effect of fast exchange between the different donor states (i.e., complete averaging during the observation time) is illustrated in Figs. 8(c) and 8(f). For the (*E*-${\left\langle \tau_{D\left(A\right)}\right\rangle }_{F}$) parameter space, a single convex static FRET-line is obtained. This line falls between the curved static FRET-lines obtained for the slow exchange and intersects the ${\left\langle \tau_{D\left(A\right)}\right\rangle }_{F}$ axis at the intensity-weighted average donor fluorescence lifetime ${\left\langle \tau_{D\left(0\right)}\right\rangle }_{F}$ = 2.71 ns. In the moment representation [Fig. 8(f)], the static FRET-line shows a higher curvature than the ideal static FRET-line (dashed gray line). Notably, even in the case of fast exchange between different donor states, the dynamic FRET-line in the moment representation remains linear (see the supplementary material, Note 5). Here, we have not considered the calculation of accurate FRET efficiencies for distributions of donor and acceptor states and instead introduced the proximity ratio. Using the general formalism introduced here, however, reference static and dynamic FRET-lines can still be defined even for uncorrected data if the corrections are instead accounted for in the FRET-lines.

Experimentally, the presence of multiple states of the donor fluorophores can be detected by a careful analysis of the donor-only population or, preferentially, a separate donor-only sample. For slow dynamics, the histogram of the molecule-wise donor fluorescence lifetimes will reveal clearly separated peaks. The FRET sample can then be analyzed in two ways. If it is possible to separate the two donor states, distinct values for the correction factor *γ* can be applied to the two populations based on the different donor quantum yields [Figs. 8(a) and 8(d)]. If such a separation is not possible, the proximity ratio, *E*_*PR*_, should be used instead of the FRET efficiency, *E*, to calculate the FRET-lines corresponding to the different photophysical states of the donor fluorophore [Figs. 8(b) and 8(e)]. For fast exchange, the presence of multiple donor states is detected from the number of lifetime components in a TCSPC analysis. In this case, a common FRET-line should be computed in the *E*_*PR*_-${\left\langle \tau_{D\left(A\right)}\right\rangle }_{F}$ parameter space as described above, since the donor states will be averaged for all FRET states [Figs. 8(c) and 8(f)].

### B. Dye-linker dynamics

**FIG. 9.**
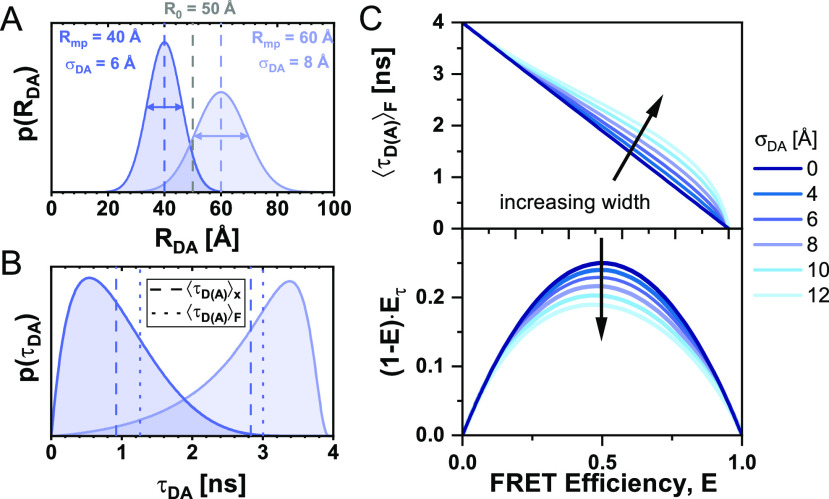
FRET-lines in the presence of fast distance fluctuations (linker dynamics). (a) and (b) The distribution of inter-dye distances ***p***(***R***_***DA***_) (a), approximated by a normal distribution as given in Eq. (60), is transformed into the corresponding distribution of donor fluorescence lifetimes ***p***(***τ***_***D***(***A***)_) (b) by the Förster relation $\tau_{D\left(A\right)}=\tau_{D\left(0\right)}{\left[1+{\left(\frac{R_{0}}{R}\right)}^{6}\right]}^{-1}$. The Förster radius, ***R***_0_, is 50 Å. (c) The broadening of the lifetime distribution causes a deviation of the static FRET-line in the $\left(E,{\left\langle \tau_{D\left(A\right)}\right\rangle }_{F}\right.$) representation toward higher values of the intensity-weighted average lifetime ${\left\langle \tau_{D\left(A\right)}\right\rangle }_{F}$. Higher values for the width of the distance distribution result in stronger deviation. In the moment representation, the contribution of the distribution width causes an inward shift of the static FRET-line. The FRET-lines in (c) are computed from the moments of the lifetime distribution as given in Eqs. (58) and (59) assuming a Gaussian distribution for the inter-dye distance as given in Eq. (60).

So far, we have assumed that a conformational state of the molecule is described by a single donor fluorescence lifetime and will be represented by a point lying on the ideal diagonal static FRET-line. A heterogeneous mixture of molecules with different FRET efficiencies, i.e., different donor–acceptor distances, would then follow this static FRET-line. This line does, however, not describe experimental data accurately. It is consistently observed that the population mean of static molecules deviates from the ideal static-FRET-line, exhibiting a bias toward longer fluorescence-weighted donor lifetimes, ${\left\langle \tau_{D\left(A\right)}\right\rangle }_{F}$. The deviation from the ideal static FRET-line is caused by the use of long flexible linkers of 10–20 Å length that tether the fluorophore to the biomolecules.^39,76,92^ Fast variations of the donor–acceptor distance *R*_*DA*_ during the observation time result in a distribution of donor lifetimes $p\left(\tau_{D\left(A\right)}\right)$ that are sampled in each single-molecule event [Figs. 9(a) and 9(b)]. Due to the finite width of the distribution, the population is, thus, shifted toward longer donor fluorescence lifetimes, whereby the deviation from the ideal static FRET-line increases with the increasing linker length and, thus, distribution width *σ*_*DA*_ [Fig. 9(c)]. Recently, we estimated that the translational diffusion coefficient of dyes tethered to proteins is on the order of 5–10 Å^2^/ns.^48^ Assuming free three-dimensional diffusion, this estimate of 10 Å^2^/ns would translate to an expected root-mean-square displacement $\left\langle x\right\rangle =\sqrt{6Dt}$ of ∼10 Å per 2 ns, resulting in significant changes of the inter-dye distance during the excited state lifetime.^93,94^ However, it is to be expected that the effective displacement is reduced due to the restriction of the dye’s movement by the linker. Under the assumption that the diffusion of the fluorophore is slow compared to the fluorescence lifetime, the fluorescence decays may be approximated by a static distribution of distances.^48,76,95^ The observation time for every single molecule on the order of milliseconds is long compared to the diffusional motion of the dyes, resulting in complete averaging of the spatial distribution of the dyes around their attachment during the observation time. Under these assumptions, we can calculate the averaged quantities and moments of the lifetime distribution based on the equilibrium distance distribution.

Different approaches for modeling the spatial distribution of tethered fluorophores have been developed.^76,96–98^ In the accessible volume (AV) approach,^92^ the possible positions of the fluorophore in the three-dimensional space are identified through a geometric search algorithm. By considering all possible combinations of donor–acceptor distances, the inter-dye distance distribution can be obtained from the accessible volumes of the donor and acceptor dyes. Extensions of the AV approach have incorporated surface trapping of fluorophores^3,48^ or accounted for the energetic contributions of linker conformation.^8,99^ More accurate models of the spatial distribution of tethered dyes are obtained by coarse-grained^48,98,100^ or all-atom^101,102^ molecular dynamics simulations, from which explicit inter-dye distance distributions may be obtained. However, as will be discussed below, the contribution of the linker flexibility is mainly defined by the width of the inter-dye distance distribution, *σ*_*DA*_, and shows only a weak dependence on the explicit shape of the distribution. Experimentally, the width of the linker distribution may be obtained from the fluorescence decay of the donor by modeling the fluorescence decays with a model function that includes a distribution of distances.^48,99^ Alternatively, by using the two-dimensional histogram of ${\left\langle \tau_{D\left(A\right)}\right\rangle }_{F}$ vs *E*, one can vary the width parameter of the static FRET-line such that it intersects with the population of static molecules. Typically, we consider a fixed standard deviation of $\sigma_{DA}∼6\;\overset{{}^{\circ}}{A}$ that satisfies benchmarking experiments on rigid DNA molecules.^39,76^

#### 1. General description in the presence of distance fluctuations

Before we discuss different models of the equilibrium distribution of inter-dye distances, we describe how the moments of the lifetime distribution can generally be calculated in the presence of distance heterogeneity. It is assumed that the fluctuations of the inter-dye distance due to the dynamics of the linkers ($\left.\tau_{linker}\right)$ are (i) slow compared to the fluorescence lifetime $\tau_{D\left(A\right)}$, leading to a distribution of lifetimes, but (ii) fast compared to the observation time *T*_obs_ (limited by the diffusion time *t*_diff_), allowing us to treat the distance distribution as stationary,

$$\tau_{D\left(A\right)}\ll \tau_{linker}\ll T_{obs}\approx t_{di\mathrm{ff}}.$$ (57)

The fluorescence lifetime of the donor, $\tau_{D\left(A\right)}$, is related to the inter-dye distance, *R*_*DA*_, by

$$\tau_{D\left(A\right)}\left(R_{DA}\right)=\tau_{D\left(0\right)}{\left[1+{\left(\frac{R_{0}}{R_{DA}}\right)}^{6}\right]}^{-1}.$$ (58)

Then, the moments of the fluorescence lifetime distribution, in terms of a distribution of inter-dye distances *p*(*R*_*DA*_), are obtained from Eq. (32) by a change of variables $\tau_{D\left(A\right)}\to \tau_{D\left(A\right)}\left(R_{DA}\right)$ given by Eq. (58),

$$\begin{array}{c} \bar{\tau_{D\left(A\right)}}=\overset{\infty }{\underset{0}{\int }}p\left(R_{DA}\right)\tau_{D\left(A\right)}\left(R_{DA}\right)dR_{DA},\\ \bar{\tau_{D\left(A\right)}^{2}}=\overset{\infty }{\underset{0}{\int }}p\left(R_{DA}\right){\left(\tau_{D\left(A\right)}\left(R_{DA}\right)\right)}^{2}dR_{DA}, \end{array}$$ (59)

which allow us to calculate the different quantities used for the representations above as a function of these two moments. In general, the distance distribution will depend on the donor–acceptor separation *R*_*mp*_ (here defined as the distance between the mean positions) and a set of parameters Λ that describe the shape of the distribution (e.g., its width). To construct the static FRET-line for a given distance distribution model *p*(*R*_*DA*_|*R*_*mp*_, Λ), we vary the mean donor–acceptor distance, *R*_*mp*_, and compute the fluorescence averaged lifetime and the FRET efficiency from the moments of the lifetime distribution given by Eq. (59). The integrals in Eq. (59) are difficult to solve analytically, even for the simple case of a normal distribution of distances, due to the sixth-power dependence between $\tau_{D\left(A\right)}$ and *R*_*D*(*A*)_. However, they can be calculated numerically for arbitrary models of the distribution. The shape of the distribution may potentially also depend on the conformation of the biomolecule and, thus, the donor–acceptor distance *R*_*mp*_, in which case the shape parameters would depend on the conformation, Λ → Λ(*R*_*mp*_). From the experimental observables *E* and ${\left\langle \tau_{D\left(A\right)}\right\rangle }_{F}$, we can only access the first and second moments of the lifetime distribution. Consequently, it is not possible to address the shape of the lifetime distribution $p\left(\tau_{D\left(A\right)}\right)$ explicitly. The same dynamic shift can thus be observed for different distributions, as long as their mean and variance (or equivalently, their first and second moments) are identical.

#### 2. FRET-lines of flexibly linked dyes

**FIG. 10.**
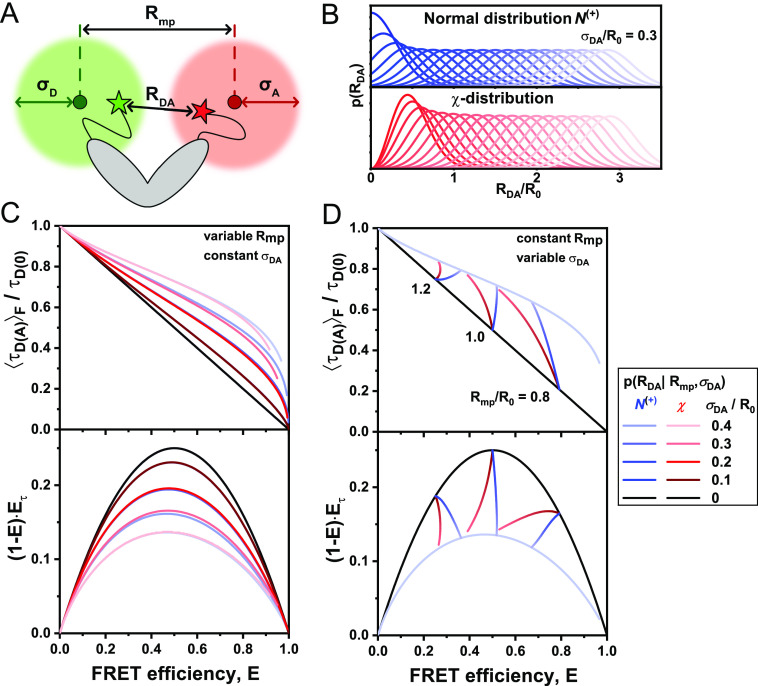
Calculation of static FRET-lines with dye linker diffusion: Difference between normal and *χ* distribution. (a) The donor and acceptor dyes are tethered to the biomolecule by flexible linkers. As a result, they can occupy an accessible volume described by the dyes’ mean position and a width parameter (*σ*_*D*_, *σ*_*A*_) that describes the linker flexibility. The distance *R*_*mp*_ describes the distance between the mean positions of the dyes, while *R*_*DA*_ is given by the instantaneous distance between the two dyes. (b) Normally distributed (top) and *χ*-distributed (bottom) inter-dye distance distribution with constant width parameter $\sigma_{DA}^{\left(l\right)}=15$ Å at $R_{0}=50$ Å. Notice that the normal distributions are truncated at small inter-dye distances. The distance distributions were calculated according to Eq. (60). (c) and (d) Linker-corrected static FRET-lines for a normal distribution (dashed lines) and *χ*-distribution (solid lines) at $\sigma_{DA}^{\left(l\right)}$ = 5, 10, 15, and 20 Å (from dark to light) and $R_{0}=50$ Å in the ($E,{\left\langle \tau_{D\left(A\right)}\right\rangle }_{F}$) (top) and moment representation (bottom). Panel (c) shows the FRET-lines for variable center distance at constant distribution width, and panel (d) shows the FRET-lines for constant center distance and variable distribution width. While the shape of the resulting FRET-lines for the normal and *χ*-distribution is similar (c), a systematic deviation is observed for the linker-averaged FRET-efficiency at increasing width parameter (d). All FRET-lines are computed from the moments of the lifetime distribution as given in Eq. (59) using the distance distributions for the inter-dye distance as given in Eq. (60), varying either the inter-dye distance (c) or the width of the distribution (d).

In practice, it is desirable to have access to simple reference static FRET-lines that can be used for graphical analysis of the measured data and the comparison of different models. To this end, an analytical model for the distance distribution is required. We first consider the simple case where the distribution of the dye positions in space follows an isotropic normal distribution [Fig. 10(a)]. This model can be interpreted as two ideal (Gaussian) chain polymer linkers that are separated by a distance *R*_*mp*_ and show no interaction with the biomolecule. In this case, the inter-dye distance vector, ***R***_*DA*_, is also normally distributed with width *σ*_*DA*_ = $\sqrt{\sigma_{D}^{2}+\sigma_{A}^{2}}$, where *σ*_*D*_ and *σ*_*A*_ are the width of the spatial distributions of the donor and acceptor dyes, respectively. The distribution of inter-dye distances, *R*_*DA*_, is then given by the non-central χ-distribution with the distance between the mean positions of the dyes, *R*_*mp*_, as the non-centrality parameter and *σ*_*DA*_ as the width parameter,

$$\begin{array}{cc} \chi \left(R_{DA}|R_{mp},\sigma_{DA}\right) & =\frac{R_{DA}}{R_{mp}}\left[N^{\left(+\right)}\left(R_{DA}|R_{mp},\sigma_{DA}\right)\right.\\ & \left.\;-N^{\left(+\right)}\left(R_{DA}|-R_{mp},\sigma_{DA}\right)\right],\\ N^{\left(+\right)}\left(R_{DA}|R_{\mu },\sigma_{DA}\right) & =\frac{1}{\sigma_{DA}\sqrt{2\pi }}e^{-\frac{1}{2}{\left(\frac{R_{DA}-R_{\mu }}{\sigma_{DA}}\right)}^{2}}with\;R_{DA}\geq 0. \end{array}$$ (60)

Here, $N^{\left(+\right)}\left(R_{DA}|R_{\mu },\sigma_{DA}\right)$ is a part of a normal distribution with a mean *R*_*μ*_ and a width *σ*_*DA*_ taken at non-negative values *R*_*DA*_ ≥ 0 (positive truncation) to avoid non-sensical distance values below zero. At small variance-to-mean ratios (i.e., at large distances), the *χ*-distribution tends to the normal distribution. Therefore, the distribution *p*(*R*_*DA*_) may be approximated by a normal distribution with mean inter-dye distance *R*_*mp*_,

$$\begin{array}{ll} p\left(R_{DA}|R_{mp},\sigma_{DA}\right) & \approx \underset{\frac{\sigma_{DA}}{R_{mp}}\to 0}{\lim}\chi \left(R_{DA}|R_{mp},\sigma_{DA}\right)\\ & =N^{\left(+\right)}\left(R_{DA}|R_{mp},\sigma_{DA}\right). \end{array}$$ (61)

As experimental distances are usually larger than 35 Å and the apparent distribution widths of the inter-dye distance are on the order of 5–10 Å, this approximation is often valid. However, for broader distributions, the truncation of the normal distribution with *R*_*DA*_ ≥ 0 results in a significant deviation from the *χ*-distribution at small inter-dye distances [see Fig. 10(b)]. Compared to the *χ*-distribution, the truncated Gaussian distance model overestimates the contribution of small distances (corresponding to high FRET efficiencies). Overall, this results only in minor deviations of the generated static FRET-lines compared to the *χ*-distribution, which are most pronounced at large distribution widths and high FRET efficiencies [Fig. 10(c)]. However, the two models show significant deviations in terms of the average FRET efficiency at identical center distances *R*_*mp*_. To illustrate this effect, we plot the change of the average FRET efficiency at constant *R*_*mp*_ and increasing *σ*_*DA*_ for the Gaussian and *χ* distance distributions in Fig. 10(c) (see solid blue and red lines, respectively). The deviation of the average FRET efficiencies between the two models increases with increasing width *σ*_*DA*_. Notably, the interpretation of average FRET efficiencies in terms of the distance between the mean positions of the dyes *R*_*mp*_ is, thus, biased by choice of the model function for the linker distribution.

In summary, the choice of the distance distribution model function has only a minor effect on the shape of the static FRET-lines, which is mainly determined by the width parameter. However, we propose that the *χ*-distribution should be preferred for the interpretation of linker-averaged FRET efficiencies in terms of physical distances when broad linker distributions are expected.

### C. Conformational dynamics in the presence of linker broadening

So far, we have only considered the effects of linker broadening for static conformations of molecules. In the presence of conformational dynamics, the total distance heterogeneity will be given by the combination of both contributions. If the timescale of the dynamics of the linkers is comparable to the timescale of conformational dynamics (e.g., for intrinsically disordered proteins), one would require a joint probability distribution of the conformational dynamics and the linker configuration. Generally, however, the dynamics of tethered dyes are much faster than the dynamics of the host molecule. It can then be assumed that the linker distribution is entirely sampled for every single molecule, allowing it to be treated as a stationary distribution for each conformational state. Consider that the conformational states are characterized by different mean donor–acceptor distances $R_{mp}^{\left(c\right)}$, which are populated with probability $p\left(R_{mp}^{\left(c\right)}|\Lambda^{\left(dyn\right)}\right)$, where Λ^(dyn)^ is the set of parameters describing the conformational dynamics, i.e., the transition rate matrix. The linker distributions in the different conformational states are given by $p\left(R_{DA}|R_{mp}^{\left(c\right)},\Lambda_{l}^{\left(c\right)}\right)$, whereby the parameters of the linker distance distribution, $\Lambda_{l}^{\left(c\right)}$, may potentially be different for the conformational states. The combined distance distribution $p\left(R_{DA}\right)$ then takes the following general form:

$$p\left(R_{DA}\right)=\int p\left(R_{DA}|R_{mp}^{\left(c\right)},\Lambda_{l}^{\left(c\right)}\right)p\left(R_{mp}^{\left(c\right)}|\Lambda^{\left(dyn\right)}\right)dR_{mp}^{\left(c\right)},$$ (62)

where the integration is performed over all possible conformational states.

We first turn to the specific case wherein we describe the linker distribution in each conformational state by a *χ*-distribution characterized by the mean inter-dye distance $R_{mp}^{\left(c\right)}$ and its corresponding width, $\sigma_{D\left(A\right)}^{\left(c\right)}$. For the case of two conformational states, the combined distribution of inter-dye distances integral in Eq. (62) then simplifies to the discrete sum

$$p\left(R_{DA}\right)=\overset{2}{\underset{c=1}{\sum }}x^{\left(c\right)}\chi \left(R_{DA}|R_{mp}^{\left(c\right)},\sigma_{DA}^{\left(c\right)}\right).$$ (63)

The dynamic FRET-line in the presence of flexible linkers is obtained by varying the species fraction *x*^(1)^ and numerically calculating the moments, as described in Eq. (59).

#### 1. Separating the contributions of linkers and conformational dynamics

The presented approach is applicable if an analytical model is available to describe the contributions of linker dynamics to the broadening of the distance distribution. In the experiment, however, we might not know the exact distribution but are able to measure the moments of the lifetime distribution in the distinct (static) conformational states experimentally. Without having to model the linker distribution explicitly, we, thus, have access to the linker-averaged moments of each conformational state *c*, ${\left\langle \tau_{D\left(A\right)}\right\rangle }_{l}^{\left(c\right)}$ and ${\left\langle \tau_{D\left(A\right)}^{2}\right\rangle }_{l}^{\left(c\right)}$, defined as

$${\left\langle \tau_{D\left(A\right)}^{\nu }\right\rangle }_{l}^{\left(c\right)}=\int \tau_{D\left(A\right)}^{\nu }\left(R_{DA}\right)p\left(R_{DA}|R_{mp}^{\left(c\right)},\Lambda_{l}^{\left(c\right)}\right)dR_{DA}.$$ (64)

For the general description of the distance distribution given in Eq. (62), the moments of the lifetime distribution in the presence of conformational dynamics are given by the double integral

$$\begin{array}{ll} {\left\langle \tau_{D\left(A\right)}^{\nu }\right\rangle }_{x} & =\int \int \tau_{D\left(A\right)}^{\nu }\left(R_{DA}\right)p\left(R_{DA}|R_{mp}^{\left(c\right)},\Lambda_{l}^{\left(c\right)}\right)\\ & \;\times p\left(R_{mp}^{\left(c\right)}|\Lambda^{\left(dyn\right)}\right)dR_{mp}^{\left(c\right)}dR_{DA}. \end{array}$$ (65)

To separate the contributions of the conformational dynamics and the linker fluctuations, we rearrange the integral to first integrate over the linker distribution, which is possible due to the separation of the timescales of the linker and conformational dynamics,

$$\begin{array}{ll} {\left\langle \tau_{D\left(A\right)}^{\nu }\right\rangle }_{x} & =\int \left[\int \tau_{D\left(A\right)}^{\nu }\left(R_{DA}\right)p\left(R_{DA}|R_{mp}^{\left(c\right)},\Lambda_{l}^{\left(c\right)}\right)dR_{DA}\right]\\ & \;\times p\left(R_{mp}^{\left(c\right)}|\Lambda^{\left(dyn\right)}\right)dR_{mp}^{\left(c\right)}\\ & =\int {\tau_{D\left(A\right)}^{\nu }}_{l}^{\left(c\right)}p\left(R_{mp}^{\left(c\right)}|\Lambda^{\left(dyn\right)}\right)dR_{mp}^{\left(c\right)}. \end{array}$$ (66)

Thus, in the calculation of the moments, we can separate the contributions of the linker distribution by first evaluating the moments of the linker distribution in each conformational state, ${\left\langle \tau_{D\left(A\right)}^{\nu }\right\rangle }_{l}^{\left(c\right)}$, which is then used to evaluate the moments in the presence of conformational dynamics. From Eq. (66), it can be shown that the variances of the linker distributions and the conformational dynamics are additive, that is,

$$\mathrm{Var}\left(\tau_{D\left(A\right)}\right)=Var^{\left(c\right)}\left({\left\langle \tau_{D\left(A\right)}\right\rangle }_{l}^{\left(c\right)}\right)+{\bar{Var^{\left(l\right)}\left(\tau_{D\left(A\right)}\right)}}^{\left(c\right)},$$ (67)

where $Var^{\left(c\right)}\left({\left\langle \tau_{D\left(A\right)}\right\rangle }_{l}^{\left(c\right)}\right)$ is the variance of the linker-averaged lifetime for all conformational states and ${\bar{Var^{\left(l\right)}\left(\tau_{D\left(A\right)}\right)}}^{\left(c\right)}$ is the average of the linker-variances over the different states [see the supplementary material, Note 3, and Sec. III E for a derivation of Eq. (67)].

The importance of these equations is that the contributions of the linkers can be treated separately from the conformational dynamics. We only require to know the linker-averaged moments, ${\left\langle \tau_{D\left(A\right)}^{\nu }\right\rangle }_{l}^{\left(c\right)}$, of the lifetime distribution of the different conformational states, which may be calculated for a particular model of the linker distance distribution [Eq. (64)] or be obtained from the observables *E* and ${\left\langle \tau_{D\left(A\right)}\right\rangle }_{F}$ of the pure states. The linker-averaged moments then replace the corresponding powers of the pure state lifetimes in the calculation of dynamic FRET-lines [Eq. (39)]. Thus, the moments of the lifetime distribution for two-state dynamic exchange, i.e., *c* ∈ {1, 2}, are given by

$$\begin{array}{c} {\left\langle \tau_{D\left(A\right)}\right\rangle }_{x}=x^{\left(1\right)}{\left\langle \tau_{D\left(A\right)}\right\rangle }_{l}^{\left(1\right)}+\left(1-x^{\left(1\right)}\right){\left\langle \tau_{D\left(A\right)}\right\rangle }_{l}^{\left(2\right)},\\ {\left\langle \tau_{D\left(A\right)}^{2}\right\rangle }_{x}=x^{\left(1\right)}{\left\langle \tau_{D\left(A\right)}^{2}\right\rangle }_{l}^{\left(1\right)}+\left(1-x^{\left(1\right)}\right){\left\langle \tau_{D\left(A\right)}^{2}\right\rangle }_{l}^{\left(2\right)}, \end{array}$$ (68)

from which the dynamic FRET-lines in the different representations are obtained by varying the species fraction *x*^(1)^ as described before. Therefore, the linearity of the dynamic mixing of the moments for conformational dynamics is still valid in the presence of linker fluctuations. Dynamic FRET-lines, thus, stay linear in the moment representation.

**FIG. 11.**
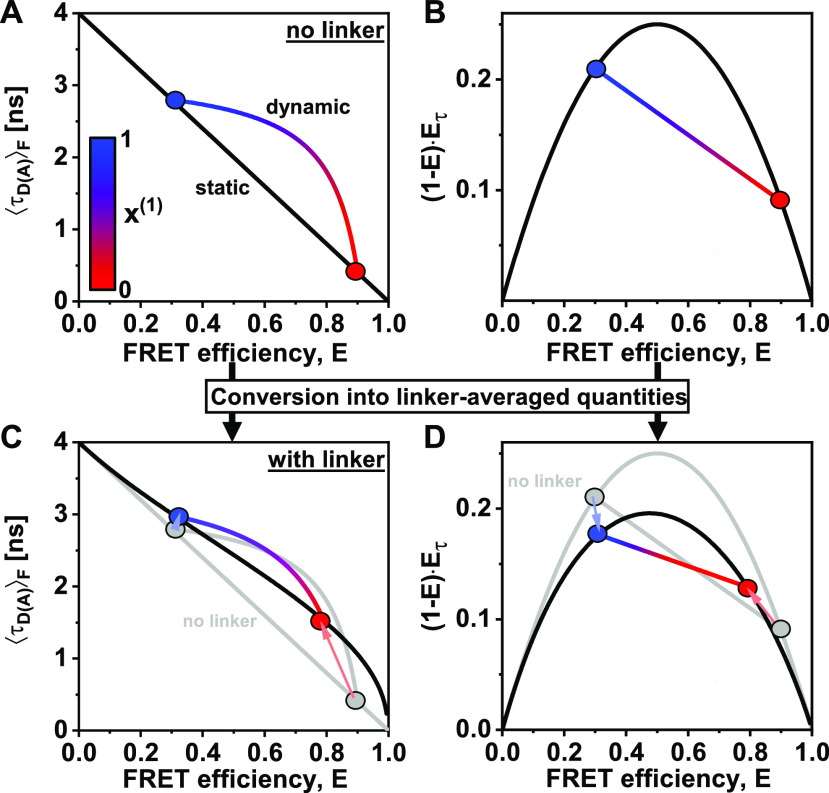
Dynamic FRET-lines in the presence of flexible linkers. (a) and (b) Static and dynamic FRET-lines in the $\left(E,{\left\langle \tau_{D\left(A\right)}\right\rangle }_{F}\right)$ parameter space (a) and in the moment representation (b) in the absence of flexible linkers. The static FRET-line is given in black, and the dynamic FRET-line is colored according to the relative contribution of the two species. (c) and (d) Static and dynamic FRET-lines in the presence of flexible linkers (black and colored lines) are shown in the $\left(E,{\left\langle \tau_{D\left(A\right)}\right\rangle }_{F}\right)$ parameter space (c) and in the moment representation (d). The FRET-lines in the absence of flexible linkers, as shown in (a) and (b), are displayed in gray. Arrows indicate the shift of the pure states after averaging over the linker distance distribution. No simple relation exists between the dynamic FRET-line in the presence and absence of flexible linkers for the $\left(E,{\left\langle \tau_{D\left(A\right)}\right\rangle }_{F}\right)$ representation (c). In the moment representation (d), the linear relationship for the dynamic exchange is retained in the presence of flexible linkers. The dynamic FRET-line is simply obtained by connecting the shifted coordinates of the pure states in the presence of flexible linkers. The curves are obtained for a donor lifetime of $\tau_{D\left(0\right)}=4$ ns, a Förster radius of ***R***_0_ = 50 Å, and interdye distances of $R_{mp}^{\left(1\right)}=57.5$ Å and $R_{mp}^{\left(2\right)}=34.5$ Å. The distribution width for the linker broadening was $\sigma_{DA}=7.5$ Å. In (a), the static FRET-line (black) is given by Eq. (22) and the dynamic FRET-line (gradient line) was calculated according to Eq. (27). In (b), the static FRET line (black) is given by Eq. (45) and the dynamic FRET-line (gradient line) was calculated according to Eq. (47). In (c) and (d), the static FRET-lines and dynamic FRET-lines were computed according to Eqs. (22) and (27) for (c) and Eqs. (45) and (47) for (d) using the linker averaged moments of the lifetime distribution as given in Eqs. (66) and (68).

**FIG. 12.**
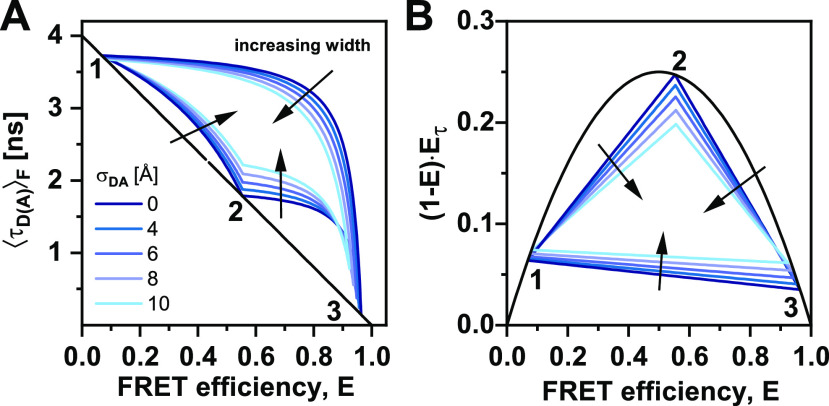
Dynamic FRET-lines in the presence of flexible linkers in three-state systems for the $\left(E,{\left\langle \tau_{D\left(A\right)}\right\rangle }_{F}\right)$ parameter space (a) and the moment representation (b). With the increasing linker width, the dynamic FRET-lines are shifted inward for both representations. In the moment representation, the linearity of the dynamic FRET-lines is retained in the presence of flexible linkers. The distances between the mean positions of the dyes, ***R***_***mp***_, for the three states are 30, 50, and 80 Å with a Förster radius ***R***_0_ = 52 Å. The static FRET-lines are given by Eq. (22) for (a) and Eq. (45) for (b). The dynamic FRET-lines were computed according to Eqs. (22) and (27) for (a) and Eqs. (45) and (47) for (b) using the linker averaged moments of the lifetime distribution, as given in Eqs. (66) and (68).

Dynamic FRET-lines in the presence of flexible linkers are illustrated in Fig. 11. In the $\left(E,{\left\langle \tau_{D\left(A\right)}\right\rangle }_{F}\right)$ representation, it is not possible to perform a simple graphical construction of the dynamic FRET-line for flexible linkers. In the moment representation, however, the dynamic FRET-line for flexible linkers is simply obtained by connecting the linker-averaged coordinates of the two states. This simplification has important implications for the accurate description of dynamic FRET-lines in complex experimental systems, where no model for the linker distribution is available. Consider, for example, the case that the width of the linker distribution depends on the inter-dye distance in an unknown manner. In this case, it is not possible to obtain a general static FRET-line. However, with the presented formalism, we only require the knowledge of the positions of the limiting static states, which are sufficient to fully describe the corresponding dynamic FRET-line. For the $\left(E,{\left\langle \tau_{D\left(A\right)}\right\rangle }_{F}\right)$ representation, the linker-averaged first and second moments of the limiting states, ${\left\langle \tau_{D\left(A\right)}\right\rangle }_{l}^{\left(i\right)}$ and ${\left\langle \tau_{D\left(A\right)}^{2}\right\rangle }_{l}^{\left(i\right)}$, can be determined from the averaged FRET observables *E* and ${\left\langle \tau_{D\left(A\right)}\right\rangle }_{F}$ of the static populations, from which the dynamic FRET-line is obtained by a linear combination of the moments [Eq. (68)] and conversion back into the $\left(E,{\left\langle \tau_{D\left(A\right)}\right\rangle }_{F}\right)$ parameter space. In the moment representation, the dynamic FRET-line is simply obtained graphically by connecting the conformational states with a straight line. Thus, for the construction of the dynamic FRET-line, it is generally not required to know the linker distance distribution in analytical form. If structural information is available, the linker distribution may also be obtained from the accessible volumes of the dyes in distinct conformations. In a three-state system, the dynamic FRET-lines in the presence of flexible linkers are shifted toward the center of the area enclosed by the limiting lines (Fig. 12).

### D. FRET-lines of flexible polymers

In Secs. IV B and IV C, we have described the contributions of the flexible linkers to the static and dynamic FRET-lines. Through the stationary distance distribution, the effects of the fast linker dynamics could be accounted for. In principle, the linkers are equivalent to short flexible polymers, which may be treated analogously to the procedure described above when a model for the equilibrium distance distribution is available. In the following, we present FRET-lines for different polymer models in the context of the potential application to the study of flexible biological polymers, such as unfolded or intrinsically disordered proteins.

#### 1. Disordered states

**FIG. 13.**
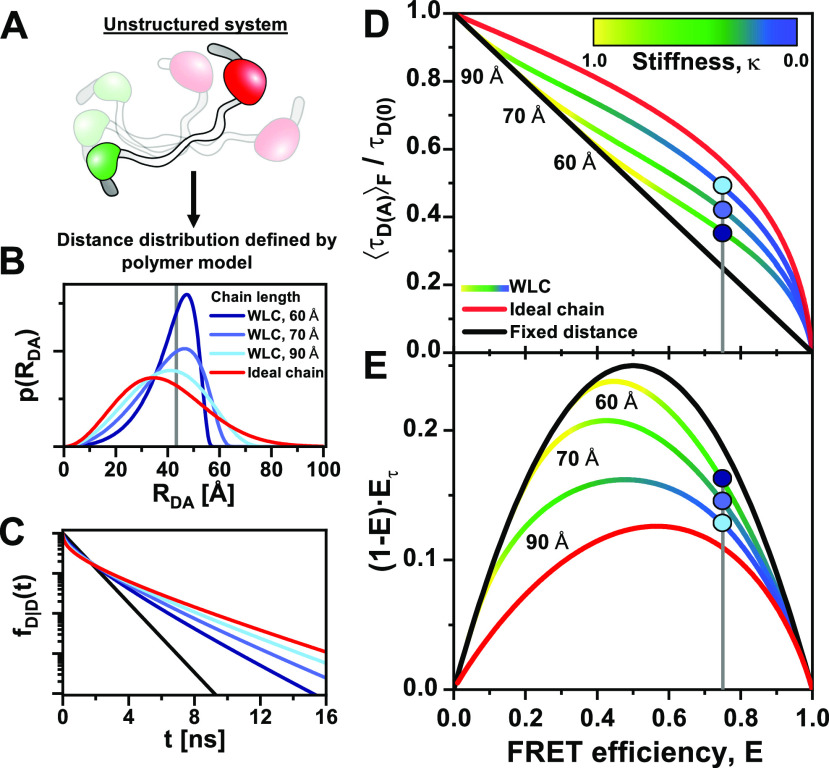
FRET-lines for disordered states. (a) Unstructured biomolecules, such as intrinsically disordered proteins, rapidly interconvert between an ensemble of structures. (b) The interdye distance distributions $p\left(R_{DA}\right)$ of an unstructured system may be described by polymer models, here given by worm-like chain (WLC) of different lengths of 60, 70, and 90 Å. The distance distributions were generated according to Eq. (69) for the ideal (Gaussian) chain and as described in the supplementary material, Note 6, for the WLC model. (c) The distance distributions define the corresponding fluorescence decays $f_{D|D}\left(t\right)$ according to Eq. (8). (d) FRET-lines for the WLC model at different polymer lengths and stiffness ***κ***. The examples shown in (a) and (b) are given as colored dots. The static FRET-line for fixed dyes is given as a black line, and the FRET-line for the Gaussian chain model is given as a red line. (e) The same data as shown in (d) in the moment representation. FRET-lines were calculated for a donor-lifetime $\tau_{D\left(0\right)}=4$ ns, and a Förster distance ***R***_0_ = 52 Å. All FRET-lines were computed from the moments of the lifetime distribution as given in Eq. (59) using the distance distributions for the inter-dye distance as given in Eq. (69) for the ideal (Gaussian) chain and in the supplementary material, Note 6, for the WLC model.

Single-molecule FRET measurements are particularly suited to characterize biomolecules with partial or lack of stable tertiary structures, such as unfolded proteins, intrinsically disordered proteins (IDPs), and proteins with intrinsically disordered regions (IDRs).^103–108^ In the one-dimensional analysis of FRET efficiency histograms, the information about the fast dynamics of these systems is hidden, and complementary methods, such as small-angle x-ray scattering (SAXS), have to be employed to assert the presence of disorder.^104–106,109^ In contrast, the knowledge of the fluorescence weighted average lifetime ${\left\langle \tau_{D\left(A\right)}\right\rangle }_{F}$ in addition to the FRET efficiency, *E* allows dynamics to be identified directly from the single-molecule FRET experiment. As described above, these quantities allow one to address the mean and variance of the distribution of fluorescence lifetimes and, thus, contain information about the mean and variance of the distribution of inter-dye distances [Figs. 13(a)–13(c)]. Here, we outline how to exploit this information to characterize IDPs and proteins with IDRs by means of FRET-lines of polymer models.

The conformational dynamics of IDPs or proteins with IDRs are usually fast compared to the observation time, with relaxation times on the order of 100 ns to several *μ*s.^110–112^ In the measurement, a single population is then observed at a position that corresponds to the average over the continuous distribution of conformations. We first consider that the disordered system is described by a Gaussian chain (GC) model. This model approximates the conformational space by a quasi-continuum of states and has previously been applied to the description of experimental single-molecule FRET histograms of *E* and ${\left\langle \tau_{D\left(A\right)}\right\rangle }_{F}$ of IDPs.^20^ The distribution of interdye distances is given by the central *χ*-distribution and depends only on the variance of the interdye distance, $\sigma_{DA}^{2}$,

$$\begin{array}{ll} p_{GC}\left(R_{DA}|\sigma_{DA}\right) & =\chi \left(R_{DA}|0,\sigma_{DA}\right)\\ & =2{\left(\frac{R_{DA}}{\sigma_{DA}}\right)}^{2}N^{+}\left(R_{DA}|0,\sigma_{DA}\right). \end{array}$$ (69)

Often, this distribution is written in terms of the mean squared distance, $\bar{R_{DA}^{2}}$, which is related to the variance by $\bar{R_{DA}^{2}}=3\sigma_{DA}^{2}$. As this model has only one variable parameter (*σ*_*DA*_), only a single Gaussian chain FRET-line may be constructed. This FRET-line describes all polymers that behave like an ideal Gaussian chain [red line in Figs. 13(d) and 13(e)]. It can be thought of as a reference line for polymers that describes how ideal the studied system behaves, analogous to the static FRET-line for structured systems. More realistically, a disordered peptide chain may be described by the worm-like chain (WLC) model (see the supplementary material, Note 6).^113,114^ The parameters defining the inter-dye distance distribution of the WLC model are the total chain length *L* and the persistence length *l*_*p*_ that define the stiffness of the chain by $\kappa =\frac{l_{p}}{L}$. In principle, the total length of the chain is known *a priori* from the protein sequence. From the experimentally observed position of the population in the two-dimensional histogram, the stiffness of the chain can then be estimated. FRET-lines for the WLC model are shown for different combinations of the parameters *κ* and *L* in Figs. 13(c) and 13(d). Notice that different combinations of *κ* and *L* can result in identical FRET efficiencies, as indicated by the horizontal line in the plot. To determine both parameters, in addition, ${\left\langle \tau_{D\left(A\right)}\right\rangle }_{F}$ needs to be known.

#### 2. Order–disorder transitions

**FIG. 14.**
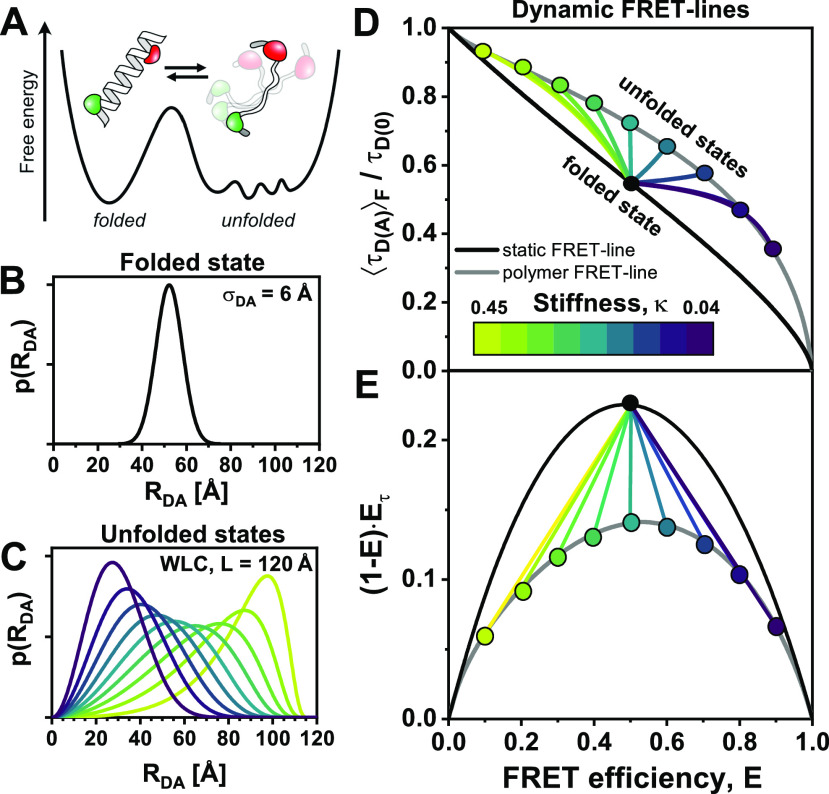
FRET-lines for order–disorder transitions. (a) Free energy landscape of a folding/unfolding transition. (b) Distance distribution of the folded state given by a non-central ***χ***-distribution as given in Eq. (60), centered at 52 Å with a width parameter of 6 Å. (c) Distance distributions $p\left(R_{DA}\right)$ for unfolded states described by the worm-like chain (WLC) model for a polymer of 120 Å length with varying stiffness ***κ*** [see the color scale in (d)]. Distance distributions were calculated as described in the supplementary material, Note 6. (d) and (e) Dynamic FRET-lines for the exchange between the folded and unfolded states in the parameter space of the experimental observables ***E*** and ${\left\langle \tau_{D\left(A\right)}\right\rangle }_{F}$ (d) and in the moment representation (e). The folded state lies on the static FRET-line (black) for fixed distances, while all unfolded states are positioned on the dashed gray line defined by the WLC model with different stiffness ***κ***. The WLC distance distributions of the unfolded state were calculated according to Ref. 113 and as described in the supplementary material, Note 6. The Förster radius is 52 Å and the donor lifetime in the absence of the acceptor is ***τ***_***D***(0)_ = 4 ns. The linker-corrected static FRET-line and the polymer FRET-line for the WLC were computed from the moments of the lifetime distribution as given in Eq. (59) using the distance distributions for the inter-dye distance as given in Eq. (60) for the folded state and the supplementary material, Note 6 for the WLC model. Dynamic FRET-lines were computed accordingly using the mixed distance distribution given in Eq. (70) by varying the species fraction of the folded and unfolded states.

Another scenario that can be identified and described by FRET-lines is the spontaneous transition between folded and unfolded states. Suppose that the distance distribution in the folded and the unfolded states is given by $p^{\left(f\right)}\left(R_{DA}|R_{mp}^{\left(f\right)}\right)$ and $p^{\left(u\right)}\left(R_{DA}|\Lambda^{\left(u\right)}\right)$, respectively. Then, the combined distance distribution is given by

$$p\left(R_{DA}\right)=x^{\left(f\right)}p^{\left(f\right)}\left(R_{DA}|R_{mp}^{\left(f\right)}\right)+\left(1-x^{\left(f\right)}\right)p^{\left(u\right)}\left(R_{DA}|\Lambda^{\left(u\right)}\right),$$ (70)

where *x*^(*f*)^ is the species fraction of the molecules in the folded state, $p^{\left(f\right)}\left(R_{DA}|R_{mp}^{\left(f\right)}\right)$ describes the linker distribution in the folded state around the average distance $R_{mp}^{\left(f\right)}$ [Fig. 14(b)], and $p^{\left(u\right)}\left(R_{DA}|\Lambda^{\left(u\right)}\right)$ describes the distance distribution in the unfolded state, dependent on the parameters of the polymer model, Λ^(*u*)^ [Figs. 14(a)–14(c)]. By varying *x*^(*f*)^ while keeping the parameters of the distance distributions [$R_{mp}^{\left(f\right)}$ and Λ^(*u*)^] constant, the dynamic FRET-line is obtained. These FRET-lines are conceptually identical to dynamic FRET-lines describing the exchange between two folded states under the assumption that the sampling of the distance distribution in the unfolded state is fast compared to the transition rate to the folded state (see Sec. IV C 1). The broad distance distribution of the unfolded state shifts the endpoint of the resulting folding FRET-line far from the static FRET-line [Figs. 14(d) and 14(e)]. Dynamic transitions between a single folded state, characterized by $R_{mp}^{\left(f\right)}$, and different unfolded states, each described by the WLC model with varying stiffness at constant length ($\left.\Lambda^{\left(u\right)}=\left\{\kappa ,L\right\}\right.$), are illustrated in Figs. 14(c) and 14(d) in the $\left(E,{\left\langle \tau_{D\left(A\right)}\right\rangle }_{F}\right)$ and moment representations. Notice how all unfolded states are described by a single curve defined by the total chain length. Even though both folded and unfolded states are described by a distribution of distances, the folding FRET-line in the moment representation remains linear [Fig. 14(d)]. Dynamic unfolding FRET-lines describe folding/unfolding transitions of proteins similar to binary dynamic FRET-lines.^75,115^ The position of the population on the folding/unfolding FRET-lines informs on kinetic rate constants of the folding/unfolding events (see Paper II). For fast-folding/unfolding transitions on the microsecond timescale, the position of the population along the folding/unfolding FRET-line may thus be used to determine the equilibrium constant of the folding process.

#### 3. Application of FRET-lines to experimental data

In this section, we review the application of FRET-lines and the moment representation to experimental data by revisiting published data on three different proteins as prototypic examples for static multi-state dynamic and disordered systems.

**FIG. 15.**
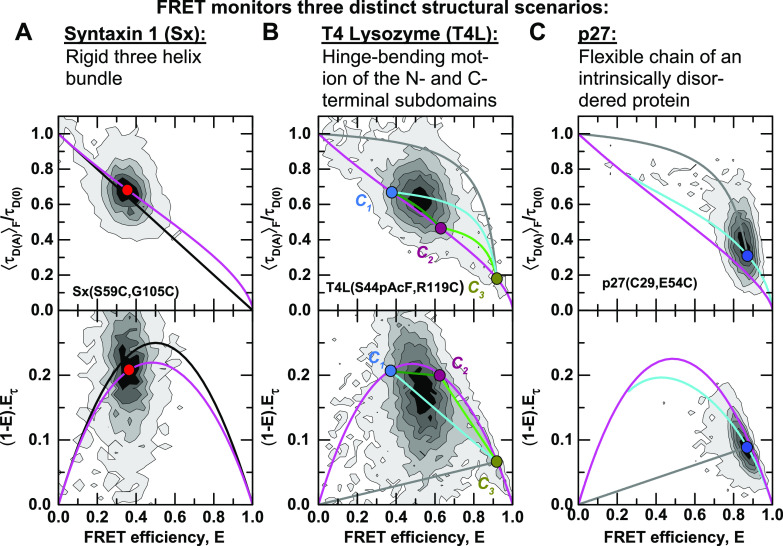
Exemplary applications of FRET-lines to experimental data. The data are shown in the *E*–${\left\langle \tau_{D\left(A\right)}\right\rangle }_{F}$ parameter space (top) and in moment representations (bottom). (a) The protein Syntaxin 1, labeled stochastically at the amino acids 59 and 105 with the dyes Alexa488 and Alexa594, shows static behavior with a single population on the linker-corrected static FRET-line (magenta, *R*_0_ = 55 Å, *σ*_*DA*_ = 6 Å).^32^ The ideal static FRET-line is shown in black. The ideal static FRET-line is given by Eq. (22), and the linker-corrected static FRET-line is computed from the moments of the lifetime distribution as given in Eqs. (58) and (59) assuming a Gaussian distribution for the inter-dye distance as given in Eq. (60). (b) The enzyme T4 lysozyme alternates between three conformational states (C_1_, blue; C_2_, purple; C_3_, ochre) during its catalytic cycle in the order C_1_ ⇌ C_2_ ⇌ C_3_.^85^ The linker-corrected static FRET-line is given in magenta (*R*_0_ = 52 Å, *σ*_*DA*_ = 6 Å). The exchange between C_1_ and C_2_ is fast (<10 *µ*s) compared to the exchange between C_2_ and C_3_ (≈230 *µ*s). Due to the fast conformational dynamics compared to the diffusion time of 1–5 ms, the population falls into the center of the triangle defined by the binary dynamic FRET-lines between the limiting states (C_1_ ⇌ C_2_, dark green; C_2_ ⇌ C_3_, light green; C_1_ ⇌ C_3_, cyan). A variant of T4L was labeled site-specifically at position S44pAcF with the dye Alexa488 and at position R119C with the dye Alexa647. The linker-corrected static FRET-line is computed from the moments of the lifetime distribution as given in Eqs. (58) and (59) assuming a Gaussian distribution for the inter-dye distance as given in Eq. (60). The dynamic FRET-lines are computed according to Eq. (47) using the linker averaged moments of the lifetime distribution as given in Eqs. (66) and (68). (c) The variant of intrinsically disordered protein p27, labeled stochastically at the cysteine residues C29 and E54C with the dyes Alexa488 and Alexa647, shows a single dynamic population that deviates from the static FRET-lines (magenta, *R*_0_ = 52 Å, *σ*_*DA*_ = 6 Å), but can be described by the WLC model with a contour length of 74 Å and a stiffness of *κ* = 0.39 (cyan).^114^ In (b) and (c), the gray line traces molecules with high FRET efficiency for which the acceptor blinked or bleached during the observation time. The linker-corrected static FRET-line is computed from the moments of the lifetime distribution as given in Eqs. (58) and (59) assuming a Gaussian distribution for the inter-dye distance as given in Eq. (60). The WLC FRET-line was computed from the moments of the lifetime distribution as given in Eq. (59) using the distance distributions for the inter-dye distance as given in the supplementary material, Note 6, by varying the stiffness.

As a first example, we consider the protein Syntaxin-1, a member of the SNARE (soluble N-ethylmaleimide-sensitive factor attachment protein receptors) family of proteins that play a central role in membrane fusion.^116^ We have previously shown that Syntaxin-1 fluctuates between a closed and open conformation with a detached SNARE motif on the sub-millisecond timescale, while the Habc domain with a three helix bundle remains stable.^32^ Placing the donor and acceptor fluorophores at different positions on this stable Habc domain, a single FRET population is observed that falls onto the linker corrected static FRET-line in both the *E*–${\left\langle \tau_{D\left(A\right)}\right\rangle }_{F}$ and moment representations [Fig. 15(a), magenta line]. Note that dye-linker correction is needed to describe the FRET population as it deviates from the ideal static FRET line [black lines in Fig. 15(a)].

As an example for multi-state dynamics, we chose the enzyme lysozyme of the bacteriophage T4 that plays an important role in phage infection by cleaving the glycosidic bonds of the saccharides of the bacterial cell wall.^117^ We have previously characterized the conformational dynamics of T4L with a hinge-bending motion of the N- and C-terminal subdomains, which alternates between an open and closed state on the microsecond timescale (states C_1_ and C_2_): Moreover, we identified a new third even more compact state in its conformational cycle that is sampled at ∼230 *µ*s (state C_3_).^85^ Accordingly, T4L shows a complex signature in the *E*–${\left\langle \tau_{D\left(A\right)}\right\rangle }_{F}$ and moment representations with a main population that deviates from the linker-corrected static FRET-line [Fig. 15(b)]. Using the interdye distances obtained from a global analysis of the fluorescence decays, we plot the expected binary dynamic FRET-lines between the three limiting states (C_1_ ⇌ C_2_, dark green; C_2_ ⇌ C_3_, and light green; C_1_ ⇌ C_3_, cyan). Clearly, the dynamic population falls in the center of the triangular region defined by the dynamic FRET-lines. It is also positioned closer to the states C_1_ and C_2_ due to the low equilibrium fraction of state C_3_ of ∼21%. Finally, we consider the dynamics of the intrinsically disordered protein p27, a member of the Kip family of cyclin-dependent kinase inhibitor proteins that plays an important role in the regulation of cell division in eukaryotes.^118^ The free form of p27 shows a single peak at high FRET efficiency in the *E*–${\left\langle \tau_{D\left(A\right)}\right\rangle }_{F}$ and moment representations that deviates from the linker-corrected static FRET-line [Fig. 15(c)].^30,114^ To describe the disorder of p27, we applied the WLC model, for which the parameters were estimated from a subensemble fluorescence decay analysis, and plot the FRET-line of the WLC model for a chain length *L* of 73.8 Å with varying stiffness *κ* (cyan line). The FRET population falls onto this line at a stiffness of *κ* = 0.39.

These three examples of structural dynamics demonstrate that FRET-lines are essential guides to identify dynamics from the correlation between intensity-based and lifetime-based FRET observables.

## V. CONCLUSIONS

FRET-lines are guides that are superimposed on the two-dimensional histograms of the FRET observables *E* and ${\left\langle \tau_{D\left(A\right)}\right\rangle }_{F}$ and provide a graphical analysis of complex kinetic networks in smFRET experiments. Here, we described a theoretical framework for FRET-lines based on a rigorous mathematical treatment and derived expressions for FRET-lines of static and dynamic molecules. In this framework, the mobility of the flexible dye linkers can be decoupled from the motion of the biomolecule, and it is readily applicable to disordered and unstructured systems. Based on the theoretical description of the experimental observables *E* and ${\left\langle \tau_{D\left(A\right)}\right\rangle }_{F}$, we propose an alternative representation based on the moments of the underlying distribution of the donor fluorescence lifetime that simplifies the data representation. In this moment representation, the static FRET-line is transformed into a parabola, while dynamic FRET-lines are linearized. This enables a graphical analysis of complex kinetic networks, which can be performed “by hand” without having to apply complex equations and provides a direct visualization of the kinetic exchange. This simplification of dynamic FRET-lines in the moment representation remains even for complex dynamics occurring in unstructured systems, such as unfolded proteins.

The application of the moment representation to experimental data reveals some limitations of the coordinate transformation from the *E*–${\left\langle \tau_{D\left(A\right)}\right\rangle }_{F}$ to the moment representation. While there is no detrimental effect on the position of different populations in the two-dimensional histograms, their width is significantly increased in the moment representation due to shot noise broadening of the experimental observables *E* and ${\left\langle \tau_{D\left(A\right)}\right\rangle }_{F}$, which is multiplied in the calculation of the moment difference. For the given examples, this effect is most evident from the broadening of the main populations for Syntaxin-1 and T4L along the y-axis [Figs. 15(a) and 15(b)]. Notably, the broadening seems to be most pronounced at low FRET efficiencies because the uncertainty of the lifetime estimate is largest for long fluorescence lifetimes.^45^

In Paper II, we focus on a quantitative analysis of the kinetics in multi-state systems by fluorescence correlation spectroscopy and fluorescence decay analysis. While FRET-lines do not consider the timescales of the dynamics explicitly, they provide important information on the connectivity of the states, which turns out to be the missing key toward finding unique solutions for the kinetics of multi-state systems.

NOMENCLATURE

**Used symbols and definitions—Theory—Experimental observables and their relations**
*E*
: FRET efficiency, calculated from the integrated photon counts
$f_{D|D}^{\left(DA\right)}$(*t*), $f_{D|D}^{\left(D0\right)}$(*t*): time-dependent fluorescence intensity or fluorescence decay of the donor after donor excitation in the presence or absence of the acceptor

$F_{D|D}^{\left(DA\right)},F_{A|D}^{\left(DA\right)},F_{D|D}^{\left(D0\right)}$

: corrected (ideal) fluorescence intensities after excitation of the donor fluorophore of the acceptor (A|D) and donor (D|D) in the presence of the acceptor (*DA*) or for a donor in the absence of FRET (D0)

$F\left(\tau_{D\left(A\right)}\right)$

: fluorescence intensity of the species with lifetime *τ*_*D*(*A*)_
*J*(*λ*): spectral overlap integral of the donor fluorescence and acceptor absorption spectrum
*k*_RET_
: rate constant of energy transfer from *D* to *A*
*k*_*F*,*D*_
: radiative rate constant of the donor fluorescence

$k_{Q}^{\left(j\right)}$

: quenching rate constant of process *j*
*n*
: refractive index of the medium
*p*(*τ*_*D*(*A*)_): distribution of fluorescence lifetimes of the donor fluorophore

$p\left(R_{DA}\right)$

: interdye distance distribution

$p_{D|D}^{\left(DA\right)}\left(t\right)$

: probability distribution of delay times for the donor after donor excitation in the presence of the acceptor
*R*_*DA*_
: donor–acceptor separation distance
*R*_0_
: characteristic distance referred to as the Förster radius

$\left\langle t\right\rangle$

: average TCSPC delay time
*t*
: TCSPC delay time

Φ _*F*,*D*_
: fluorescence quantum yield of the donor, *D*
*κ* ^2^
: orientation factor for the transition dipoles of the FRET dyes

$\tau_{D\left(A\right)},\tau_{D\left(0\right)}$

: donor fluorescence lifetime in the presence and absence of the acceptor

${\left\langle \tau_{D\left(A\right)}\right\rangle }_{F}$

: intensity-averaged donor fluorescence lifetime
${\left\langle \tau_{D\left(A\right)}\right\rangle }_{x}$, ${\left\langle \tau_{D\left(0\right)}\right\rangle }_{x}$: species-averaged donor fluorescence lifetime in the presence and absence of the acceptor
$\bar{\tau_{D\left(A\right)}}$, $\bar{\tau_{D\left(A\right)}^{2}}$: first and second moments of the distribution of donor fluorescence lifetimes
*τ*_*MLE*_
: lifetime estimate obtained from the maximum likelihood estimation

**Comparison between FRET-lines and intensity-based approaches**
*E*_*i*_
: sample obtained for the FRET efficiency within a single-molecule event in BVA
N
: number of photons used for the sampling window to estimate *σ*_*E*_ in BVA
*σ*_*E*_
: BVA standard deviation of the FRET efficiency within a single-molecule event

**FRET-lines of static and dynamic molecules**
ds: dynamic shift, defined as the maximum deviation of the dynamic FRET-line orthogonal to the static FRET-line
*E* ^(*i*)^
: FRET efficiency of species *i*
*k*_*ij*_
: microscopic interconversion rate constant from state *j* to state *i*
*x* ^(*i*)^
: species-fraction of species *i*

$\delta \left(x\right)$

: Dirac delta function

$\tau_{D\left(A\right)}^{\left(i\right)}$

: pure-state donor lifetime of species *i*

${\left\langle \tau_{D\left(A\right)}^{2}\right\rangle }_{x}$

: species-averaged squared donor fluorescence lifetime

$\tau_{D\left(A\right)}^{\left(i\right)}$

: pure-state donor lifetime of species *i*

**General definition of FRET-lines**

$p\left(E,{\left\langle \tau_{D\left(A\right)}\right\rangle }_{F}|\lambda ,\Lambda_{f}\right)$

: conditional distribution of the experimental observables *E* and ${\left\langle \tau_{D\left(A\right)}\right\rangle }_{F}$, given *λ* and Λ_*f*_

$p\left(\tau_{D\left(A\right)}|\lambda ,\Lambda_{f}\right)$

: conditional distribution of donor fluorescence lifetimes, given *λ* and Λ_*f*_
Λ, *p*(Λ): set of parameters describing the experiment and model and their probability
Λ_*f*_: parameters that are fixed for the FRET-line
*λ*
: variable parameter used for the generation of FRET-line

$\bar{\tau_{D\left(A\right)}^{\nu }}\left(\lambda ,\Lambda_{f}\right)$

: *v*th moment of the lifetime distribution, given *λ* and Λ_*f*_ (*v* = {1, 2})

**Moments of the lifetime distribution and alternative representations**
$\mathrm{Var}\left(\tau_{D\left(A\right)}\right)$, Var(*E*): variance of the donor fluorescence lifetime or FRET efficiency

$Var^{\left(c\right)}\left(E\right)$

: contribution of conformational dynamics to the variance of the FRET efficiency
Γ: difference between the normalized first and second moments of the lifetime distribution

$\sigma_{SN}^{2}$

: contribution of shot noise to the variance of the FRET efficiency

**FRET-lines in the presence of linker dynamics**

$N^{\left(+\right)}\left(R_{DA}|R_{mp},\sigma_{DA}\right)$

: positive-truncated normal distribution of inter-dye distance *R*_*DA*_
*R*_*mp*_
: distance between mean dye positions

$R_{DA}^{\left(c\right)},R_{mp}^{\left(c\right)}$

: interdye distance and distance between mean dye positions in the conformational state *c*
*T*_obs_
: observation time of a single-molecule event

$\Lambda_{l}^{\left(c\right)}$

: linker parameters describing the inter-dye distance distribution in the conformational state *c*
Λ^(dyn)^: parameters describing the conformational dynamics (transition rate matrix)
*σ*_*DA*_
: width parameter of the inter-dye distance distribution
*σ*_*D*_, *σ*_*A*_: width of the positional distributions of the donor or acceptor fluorophore

$\sigma_{DA}^{\left(c\right)}$

: linker distribution width in conformation *c*

${\left\langle \tau_{D\left(A\right)}^{\nu }\right\rangle }_{l}^{\left(c\right)}$

: *ν*th linker-averaged moment of the fluorescence lifetime of the conformational state *c*
*τ*_linker_
: characteristic timescale of linker fluctuations

$\chi \left(R_{DA}|R_{mp},\sigma_{DA}\right)$

: *χ*-distribution of inter-dye distance *R*_*DA*_

**FRET-lines for flexible polymers**
*l*_*p*_
: persistence length of the chain
*L*
: length of the chain

$\bar{R_{DA}^{2}}$

: mean squared interdye distance used in the Gaussian chain polymer model
*κ*
: stiffness of the chain

## SUPPLEMENTARY MATERIAL

See the supplementary material for an extensive description of the general model for FRET-lines discussed in Sec. III D, the derivation of the equation for the dynamic shift as given in Sec. III C in Eq. (28), a detailed treatment of the effect of multiple photophysical states of the donor as described in Sec. IV A, a proof of Eq. (49) in Sec. III G on the geometric estimation of species fractions, a proof that the experimental lifetime estimate corresponds to the intensity-weighted average fluorescence lifetime as discussed in Sec. II D, and the distance distribution function for the worm-like chain model.

## Data Availability

The data that support the findings of this study are available from the corresponding authors upon reasonable request. To support the wide use FRET-lines presented in this Tutorial, we provide extensive software to generate the lines for different user levels. Computational tools for the calculation of FRET-lines discussed in this work in the Python programming language are available at https://github.com/Fluorescence-Tools/FRETlines, Ref. 120. The repository includes example Jupyter notebooks as direct tutorials on how to generate the different FRET-lines step-by-step by an interactive exploration of static, dynamic, and the different polymer FRET-lines. As a second tool, we provide a graphical user interface in the program “FRET-lines explorer” that is available with the software package for multiparameter fluorescence spectroscopy available at https://www.mpc.hhu.de/software/mfd-fcs-and-mfis, Ref. 121, and separately as the supplementary material.
